# The genetic basis of drought tolerance in the high oil crop *Sesamum indicum*

**DOI:** 10.1111/pbi.13100

**Published:** 2019-03-05

**Authors:** Komivi Dossa, Donghua Li, Rong Zhou, Jingyin Yu, Linhai Wang, Yanxin Zhang, Jun You, Aili Liu, Marie A. Mmadi, Daniel Fonceka, Diaga Diouf, Ndiaga Cissé, Xin Wei, Xiurong Zhang

**Affiliations:** ^1^ Oil Crops Research Institute of the Chinese Academy of Agricultural Sciences Key Laboratory of Biology and Genetic Improvement of Oil Crops Ministry of Agriculture Wuhan Hubei China; ^2^ Centre d'Etude Régional pour l'Amélioration de l'Adaptation à la Sécheresse (CERAAS) Thiès Sénégal; ^3^ Laboratoire Campus de Biotechnologies Végétales Département de Biologie Végétale Faculté des Sciences et Techniques Université Cheikh Anta Diop Dakar Sénégal; ^4^ College of Life Sciences Shanghai Normal University Shanghai China

**Keywords:** candidate genes, drought tolerance, functional alleles, genome‐wide association study, *Sesamum indicum*, *SiSAM*

## Abstract

Unlike most of the important food crops, sesame can survive drought but severe and repeated drought episodes, especially occurring during the reproductive stage, significantly curtail the productivity of this high oil crop. Genome‐wide association study was conducted for traits related to drought tolerance using 400 diverse sesame accessions, including landraces and modern cultivars. Ten stable QTLs explaining more than 40% of the phenotypic variation and located on four linkage groups were significantly associated with drought tolerance related traits. Accessions from the tropical area harboured higher numbers of drought tolerance alleles at the peak loci and were found to be more tolerant than those from the northern area, indicating a long‐term genetic adaptation to drought‐prone environments. We found that sesame has already fixed important alleles conferring survival to drought which may explain its relative high drought tolerance. However, most of the alleles crucial for productivity and yield maintenance under drought conditions are far from been fixed. This study also revealed that pyramiding the favourable alleles observed at the peak loci is of high potential for enhancing drought tolerance in sesame. In addition, our results highlighted two important pleiotropic QTLs harbouring known and unreported drought tolerance genes such as *SiABI4*,* SiTTM3, SiGOLS1*,* SiNIMIN1* and *SiSAM*. By integrating candidate gene association study, gene expression and transgenic experiments, we demonstrated that *SiSAM* confers drought tolerance by modulating polyamine levels and ROS homeostasis, and a missense mutation in the coding region partly contributes to the natural variation of drought tolerance in sesame.

## Introduction

Sesame (*Sesamum indicum* L., 2n = 26) is a traditional oilseed crop with one of the highest oil contents and qualities amongst the major oil crops. Its seeds contain almost 18% protein and the oil is rich in polyunsaturated fatty acids (PUFA) and natural antioxidants such as sesamin, sesamolin and tocopherol homologues (Anilakumar *et al*., 2010). The numerous beneficial effects of sesame oil on human health along with the cosmetic and engineering applications have promoted the steep increase in sesame seed demand (Wang *et al*., 2012). Over the last decade, the production of sesame seeds has increased more than twice and its sale price has nearly tripled (FAOSTAT, 2017).

Sesame is a resilient crop with a strong adaptation to drought‐prone environments. As compared to other major food crops, sesame better survives drought (Islam *et al*., 2016). However, it remains particularly sensitive to drought occurring during the germination and flowering stages (Boureima *et al*., 2011; Sun *et al*., 2010). In fact, sesame is grown in arid and semi‐arid areas characterized by high temperatures, high levels of solar radiation, high evaporative demand and unpredictable drought episodes which greatly impair the productivity (Hassanzadeh *et al*., 2009; Witcombe *et al*., 2007). Several traits of the plant have been reported to be affected by drought stress including the germination rate, plant growth, flowering, number of capsules per plant, seed yield as well as oil yield and quality (Bahrami *et al*., 2012; Boureima *et al*., 2016; Hassanzadeh *et al*., 2009; Kassab *et al*., 2012; Sun *et al*., 2010). As a consequence, sesame seed yields are generally low (300–400 kg/ha) in most of the arid and semi‐arid areas (Islam *et al*., 2016).

A key step towards developing drought tolerant sesame varieties is the elucidation of the genetic basis of tolerance to water stress in this species. Unfortunately, till date, very few molecular studies have been performed to decipher sesame response to drought (Dossa, 2016) and no QTL or functional marker associated with drought has been reported. Hence, it is crucial to develop genomic resources that can be employed for the improvement of drought tolerance in sesame varieties (Dossa *et al*., 2017). In general, traits that contribute to drought tolerance in plants are generally quantitative and involve multiple genes (Kang *et al*., 2015). According to Juenger (2013), variation in drought tolerance is almost always found in a large collection of accessions. Sesame harbours a huge diversity that is probably linked to its cultivation in a large range of environments coupled with long‐term natural and artificial selections (Bedigian and Harlan, 1986; Wei *et al*., 2015). Understanding the genetic basis of phenotypic variation for drought tolerance can help to efficiently utilize these wealthy genetic resources for sesame improvement.

Genome‐wide association study (GWAS) has proven to be a powerful approach in both humans and plants for detecting genes underlying variation in a natural population (Huang and Han, 2014). GWAS is based on genetic linkage disequilibrium (LD) and takes full advantage of natural variation and ancient recombination events (Nordborg and Weigel, 2008). GWAS has been carried out successfully for dissecting complex trait loci such as flowering time, leaf angle, leaf size, disease resistance, plant architecture, drought tolerance, yield, oil content and quality related traits in many crops, including maize, rice, sorghum, cotton, peach and *Brassica napus* (Cao *et al*., 2016; Guo *et al*., 2018; Huang *et al*., 2010; Li *et al*., 2013, 2016; Lu *et al*., 2017; Morris *et al*., 2012; Su *et al*., 2016; Tian *et al*., 2011). The release of a high quality reference genome of sesame has opened the door for dissecting the genetic architecture of complex agronomic traits (Wang *et al*., 2014). In this regard, 705 diverse sesame accessions collected worldwide were re‐sequenced on the Illumina HiSeq 2000 platform and employed for the first time in a comprehensive GWAS for oil‐related traits (Wei *et al*., 2015). This fully sequenced population represents tremendous and inestimable genome‐wide information for further implementations of GWAS on various key agronomic traits in sesame, including drought tolerance.

In this study, the natural variation amongst 400 sesame accessions in drought tolerance was exploited to perform a large‐scale GWAS on five traits related to drought tolerance, which can be classified into two groups: plant survival related traits and productivity and yield maintenance related traits under drought stress. To better reflect drought tolerance, we mainly focused on drought tolerance index data (the ratio of the trait value under stress to the trait value before stress; Guo *et al*., 2018). This led to the identification of key genomic regions and loci underlying drought tolerance in sesame. In addition, by integrating functional genome annotation, gene expression, bioinformatics, yeast and *Arabidopsis* genetic transformation analyses, several candidate genes for drought tolerance were discovered in sesame, mostly as unreported drought tolerance genes. Overall, our results shed light on the genetic basis of drought tolerance in sesame and lay the foundation for marker‐assisted breeding efforts to develop highly drought tolerant varieties.

## Results

### SNP distribution on the linkage groups and linkage disequilibrium

The genotypic dataset resulting from the data cleaning pipeline and imputation was composed of 1 000 939 SNPs covering all the 16 linkage groups (LGs) of the sesame genome. The LG14 encompassed the lowest number of SNPs (23 371) while the LG3 had the highest number (103 814). The average number of SNPs per LG was 62 559 and the SNP density ranged from 3 to 7 SNPs/kb with an average 5 SNPs/kb (Table S1). Based on the genotypic dataset, the *r* ^2^ estimate of linkage disequilibrium (LD) reached half of its initial value (0.55) at a distance of 88 kb and went below 0.2 at distances above 200 kb (Figure S1A). Given this moderate level of LD decay rate in sesame, the average density of 5 SNPs/kb is fully sufficient to reach a good resolution for whole genome association mapping.

### Population structure

We explored the population structure of the 400 sesame accessions based on a neighbour‐joining tree and identified two main groups of accessions (Figure S1B and Table S2). There was no obvious evidence of consistency between origins of the accessions and the grouping patterns from the phylogenetic tree. Accessions from the group coloured in red were mainly from the northern areas whereas the second group coloured in green gathered 292 sesame accessions from several countries in the tropical area (Figure S1C). These results were further confirmed by the output of the STRUCTURE software (Figure S1D). Furthermore, the SNP dataset was employed for Principal Component Analysis (PCA). The first two axes (F1 and F2) of the PCA explained 23% of the total genetic variation and similarly as the neighbour‐joining tree, two recognizable groups of accessions were obtained (Figure S1E). We computed *F*_st_ index between these two groups to examine their level of genome‐wide genetic differentiation. A very weak *F*_st_ index of 0.036 was observed between the two groups. The weak population structure, coupled with a high marker density and a moderate LD decay rate may offer ample power and resolution for sesame GWAS in this study.

### Phenotypic analysis of drought tolerance related traits in the association panel

In this study, we investigated five traits, including stem length (SL), survival rate (SR), wilting level (WL), capsule number (CN) and seed yield (Yie) in control and stress conditions. Although the plant water status parameters are important in deciphering drought tolerance, we did not include them in this work. In fact, plant water status parameters are highly dynamic and vary within minutes (Tardieu *et al*., 2017). Given the size of the population (over 7200 plants each year), investigating accurately these parameters would require a sophisticated automated platform, unfortunately, we do not have.

**Figure 1:**
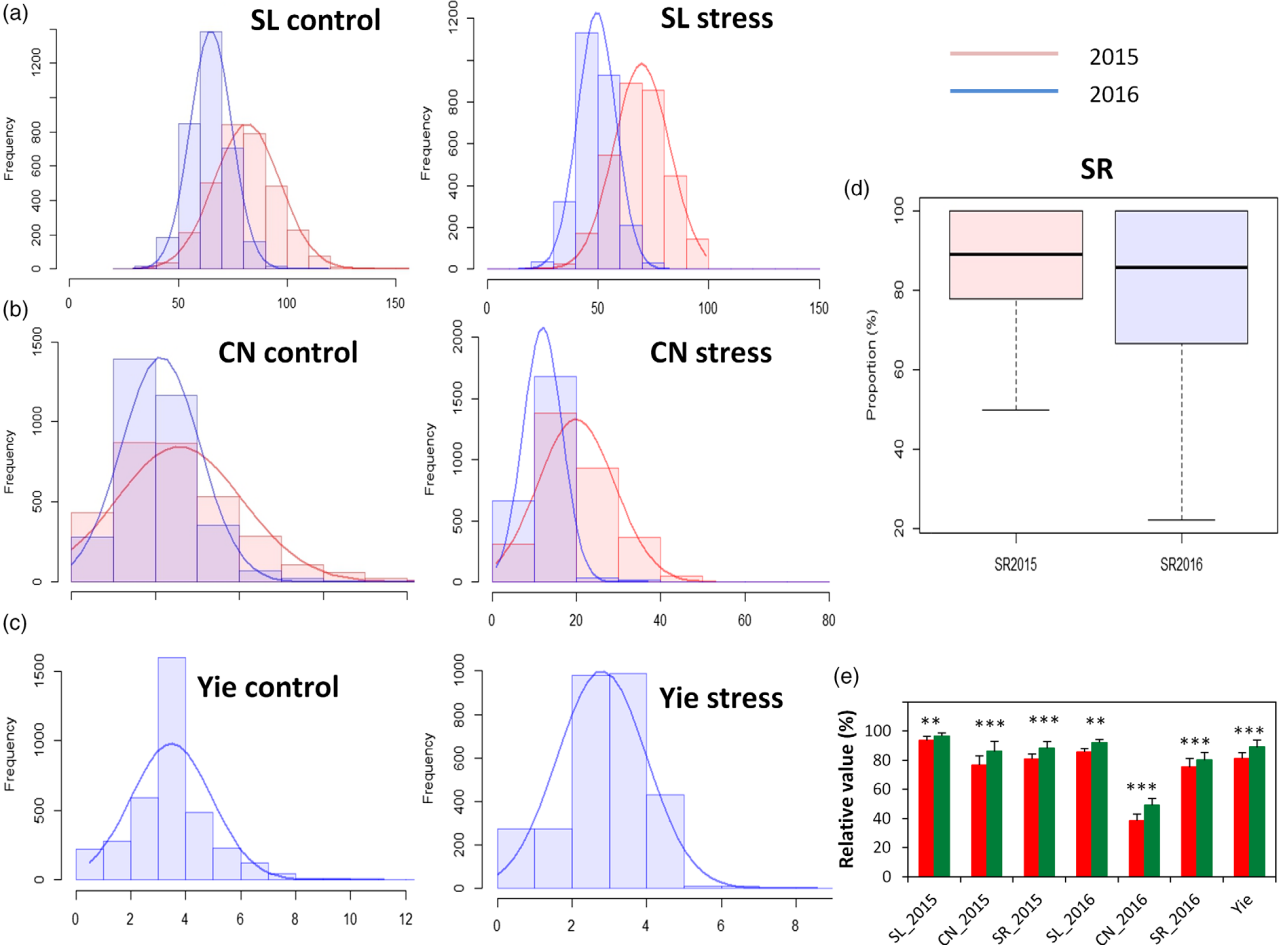
Frequency distribution and boxplot of the mean values for drought tolerance traits in 400 *Sesamum indicum* accessions under control and water stress conditions during 2015 and 2016. (a) stem length (SL) in control and water stress. (b) Capsule number (CN) in control and water stress. (c) Seed yield (Yie) in control and water stress. (d) Survival rate (SR) of the plants. (e) Comparison of drought tolerance indexes between the subpopulation from the tropical area (coloured in green, 292 accessions) and the subpopulation from the northern area (coloured in red, 108 accessions). **, *** represent a significant difference between the two groups at *P* < 0.01, 0.001, respectively.

Extensive phenotypic variations for SL, Yie, SR and CN were observed in the sesame association panel (Figure 1a–d, Table 1). SL, Yie and CN traits exhibited normal distributions. SL was the most stable trait in both control and stress conditions ranging from 14 to 120 cm, with a coefficient of variation (CV) ranging from 14.53% to 18.54%. In contrast, Yie ranged from 0.01 to 11.21 g and displayed the highest CV ranging from 66.62% to 76.92%. In general, control plants performed better than the stressed plants and CN was the most affected trait by drought stress in the 2 years. Also, the drought stress effects on the assayed traits were more striking in 2016 as compared to 2015. This is consistent with the analysis of variance (ANOVA), which indicated that the accession (A), year (Y) and interactions between the accession and year (A × Y) were significant for all traits (*P* < 0.001). In addition, relatively high (61.67%–72.61%) broad‐sense heritability (*H* ^2^) was estimated for SL and CN in both control and stress conditions. Based on the drought tolerance indexes, we observed that accessions from the tropical area displayed significantly higher drought tolerance than those from the northern area (*P* < 0.01) (Figure 1e).

**Table 1:** Phenotypic variation in drought tolerance‐related traits of the *Sesamum indicum* association panel under control and water stress conditions in 2015 and 2016

Trait	Year	Treatment	Mean ± SD	Range	CV (%)	Skewness	Kurtosis	A	Y	A*Y	*H* ^2^ (%)
SL	2015	Control	81.61 ± 15.12	29–120	18.54	0.30	0.98	***	*	*	72.05
		Stressed	69.11 ± 13.10	17–99	16.35	−0.06	0.51				72.61
	2016	Control	70.93 ± 9.50	29–119	14.63	0.15	0.54				
		Stressed	60.43 ± 8.64	14–82	14.53	0.14	0.57				
CN	2015	Control	25.68 ± 6.11	1–97	58.83	0.97	1.46	***	*	*	61.98
		Stressed	19.80 ± 4.69	1–53	59.06	1.06	2.76				61.67
	2016	Control	21.20 ± 5.34	1–89	43.99	1.13	4.21				
		Stressed	12.92 ± 3.41	1–37	60.82	0.22	0.03				
SR	2015	Stressed	87.92 ± 8.48	0–100	18.75	−1.98	5.04				
	2016	Stressed	78.96 ± 8.97	0–100	27.83	−1.14	1.01				
Yie	2016	Control	3.4 ± 1.6	0.5–11.21	66.62	1.00	1.19	***			
	2016	Stressed	2.32 ± 1.0	0.01–8.57	76.92	1.56	4.44	***			

A*Y, interaction of accessions and year; A, accessions; CN, capsule number; CV (%), coefficient of variation; *H* ^2^ (%), broad‐sense heritability; SD, standard deviation; SL, stem length; SR survival rate; Y, Year; Yie, seed yield.

Symbols *,*** represent a significant difference at *P* < 0.05, 0.001, respectively.

### Identification of significant signals associated with drought tolerance indexes

To investigate the genetic variants governing drought tolerance in sesame, GWAS were performed independently using drought tolerance index data (ratio of the trait value under drought stress condition to the trait value under normal condition) from the 2 years (Long *et al*., 2013). A total of 569 significant loci (*P* < 7.8 × 10^−6^) was identified for the five drought tolerance indexes. These loci were distributed mostly in clusters across ten LGs. Regions of 88 kb (corresponding to the LD window) upstream and downstream of the peaks and harbouring at least two clustered significant loci were defined as quantitative trait loci (QTL). The CN, wilting level (WL) and SL related indexes displayed the lowest numbers of significant signals for both years. Conversely, Yie and SR related indexes involved hundreds of clustered significant loci, indicating a complex genetic architecture of these traits. The full list of significant signals detected in the 2 years is detailed in Table S3.

**Figure 2:**
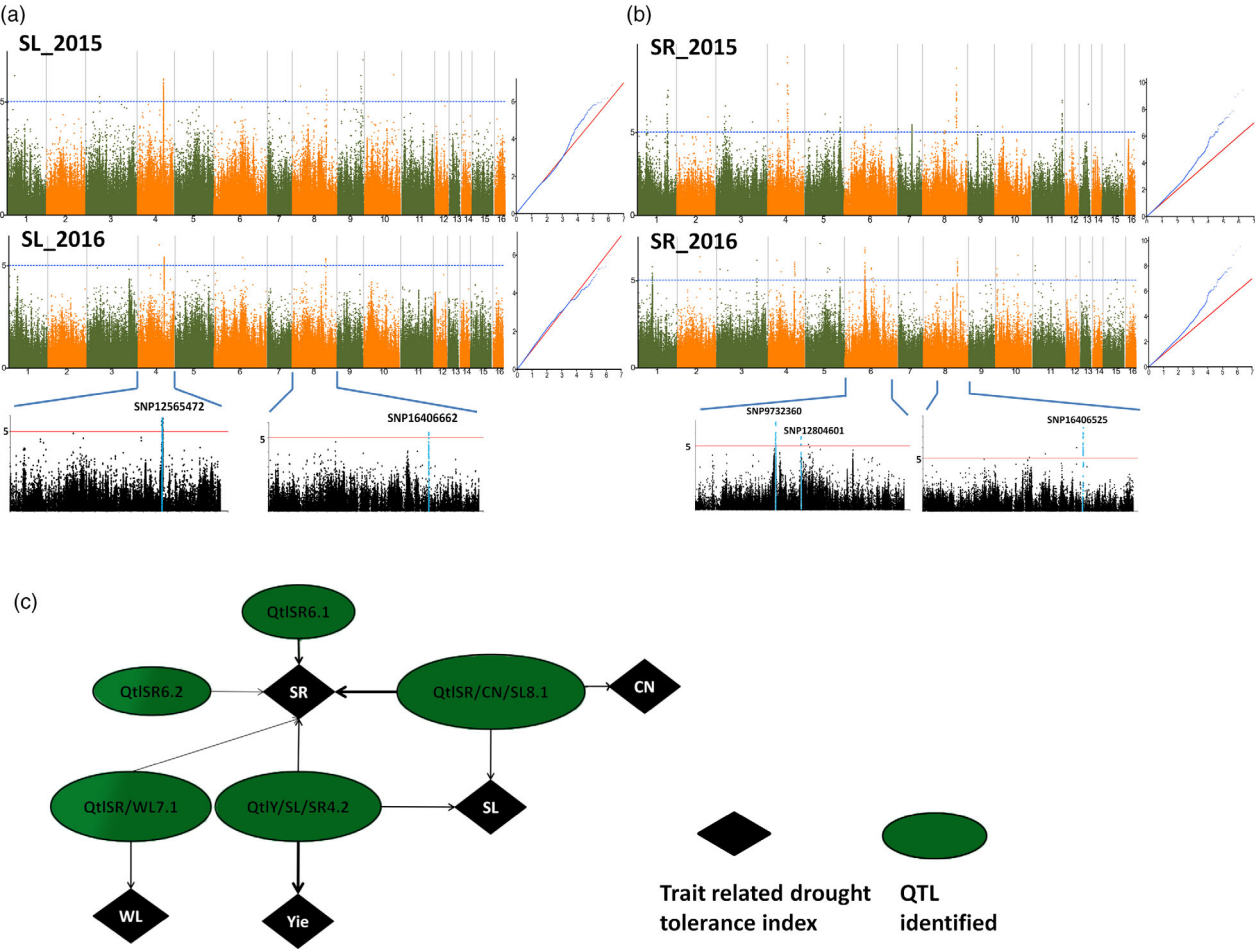
Significant loci and quantitative trait loci (QTLs) associated with SL index and SR detected by the genome‐wide association study using the Mixed model in *Sesamum indicum* during 2015 and 2016. (a) Manhattan plot and QQ plot of SL. The significant trait‐associated QTLs constitutively identified on LG4 and LG8 for the 2 years are highlighted in blue in the regional plots with the names of the lead locus placed on the top. (b) Manhattan plot and QQ plot of SR. The significant trait‐associated QTLs constitutively identified on LG6 and LG8 for the 2 years is highlighted in blue in the regional plots with the names of the lead locus placed on the top. (c) The network based on shared QTLs between different drought tolerance indexes in sesame. The width of the arrow depicts the amplitude of the QTL contribution to the trait.

In this study, we further focused on the most reliable and stable QTLs that were constitutively identified in the 2 years or associated with various traits. In total, 140 loci were identified and resolved to ten QTLs each led by a peak (Figure 2, Figure [Link], [Link], [Link]). The 10 peaks (−log_10_ (P) values ranging between 5.2 and 8.86) were located on LG4, LG6, LG7 and LG8. Phenotypic variance explained (PVE) values of the GWAS peaks ranged between 2.21% and 10.70%. The locus SNP16409277 associated with the CN index has the lowest contribution whereas the loci SNP12606000 and SNP16406525 strongly contribute to the Yie and SR indexes, respectively (Table 2). Collectively, the peak loci can explain over 40% of the phenotypic variation in sesame. Within the ten stable QTLs, eight were co‐detected for various traits, suggesting that they are pleiotropic QTLs. One QTL (QtlCN8.1~QtlSR8.1~QtlSL8.1) located on LG8 was co‐associated with CN, SR and SL indexes. Another QTL (QtlY4.1~QtlSR4.2~QtlSL4.1) located on LG4 was commonly identified for Yie, SR and SL indexes. Furthermore, we detected a shared QTL (QtlSR7.1~ QtlWL7.1) between WL and SR, which may be essential for plant survival and growth under drought conditions (Figure 2c).

**Table 2:** SNPs significantly and constitutively associated with drought tolerance traits in *Sesamum indicum*

Trait	LG	QTL name	SNP	−log10 (P)	refbase	SNPbase	MAF	Genes in LD	PVE (%)	Candidate gene ID	Gene name
CN	8	QtlCN8.1	SNP16409277	5.77	A	**C**	0.058	30	2.21‐3.32	SIN_1022782*	SiINIMIN1
										SIN_1022789*	SiSAM
										SIN_1022774	SiGOLS1
SL	4	QtlSL4.1	SNP12565472	5.99	C	**A**	0.051	19	4.64‐6.75	SIN_1012134*	SiTTM3
	8	QtlSL8.1	SNP16406662	5.22	G	**A**	0.058	30	2.85‐4.11	SIN_1022782*	SINIMIN1
										SIN_1022789*	SiSAM
										SIN_1022774	SiGOLS1
SR	4	QtlSR4.2	SNP12606000	5.62	C	**A**	0.051	18	3.72	SIN_1012139*	SiABI4
										SIN_1012134	SiTTM3
	6	QtlSR6.1	SNP9732360	6.84	**A**	G	0.136	18	4.50‐7.52	SIN_1015691*	SiP450
	6	QtlSR6.2	SNP12804601	5.68	**A**	G	0.064	12	3.12‐7.42	SIN_1015693*	NA
										SIN_1005662	SiPOD3
	7	QtlSR7.1	SNP7867981	5.2	**G**	A	0.402	23	4.18	SIN_1004723*	NA
										SIN_1004716	NA
	8	QtlSR8.1	SNP16406525	8.86	**T**	C	0.054	30	8.91‐10.70	SIN_1022782*	SiNIMIN1
										SIN_1022789*	SiSAM
										SIN_1022774	SiGOLS1
WL	7	QtlWL7.1	SNP7867981	5.93	**G**	A	0.402	23	5.15‐6.32	SIN_1004723*	NA
										SIN_1004716	NA
Yie	4	QtlY4.1	SNP12606000	8.04	C	**A**	0.051	18	10.34	SIN_1012139*	SiABI4
										SIN_1012134*	SiTTM3

PVE (%), phenotypic variance explained during the 2 years; refbase, reference allele; SNPbase, mutated allele; MAF, minor allele frequency.

* Genes containing significant SNPs; alleles in bold represent the favourable alleles.

### Mining of favourable alleles at the peaks and their pyramiding effect on drought tolerance in sesame

**Figure 3:**
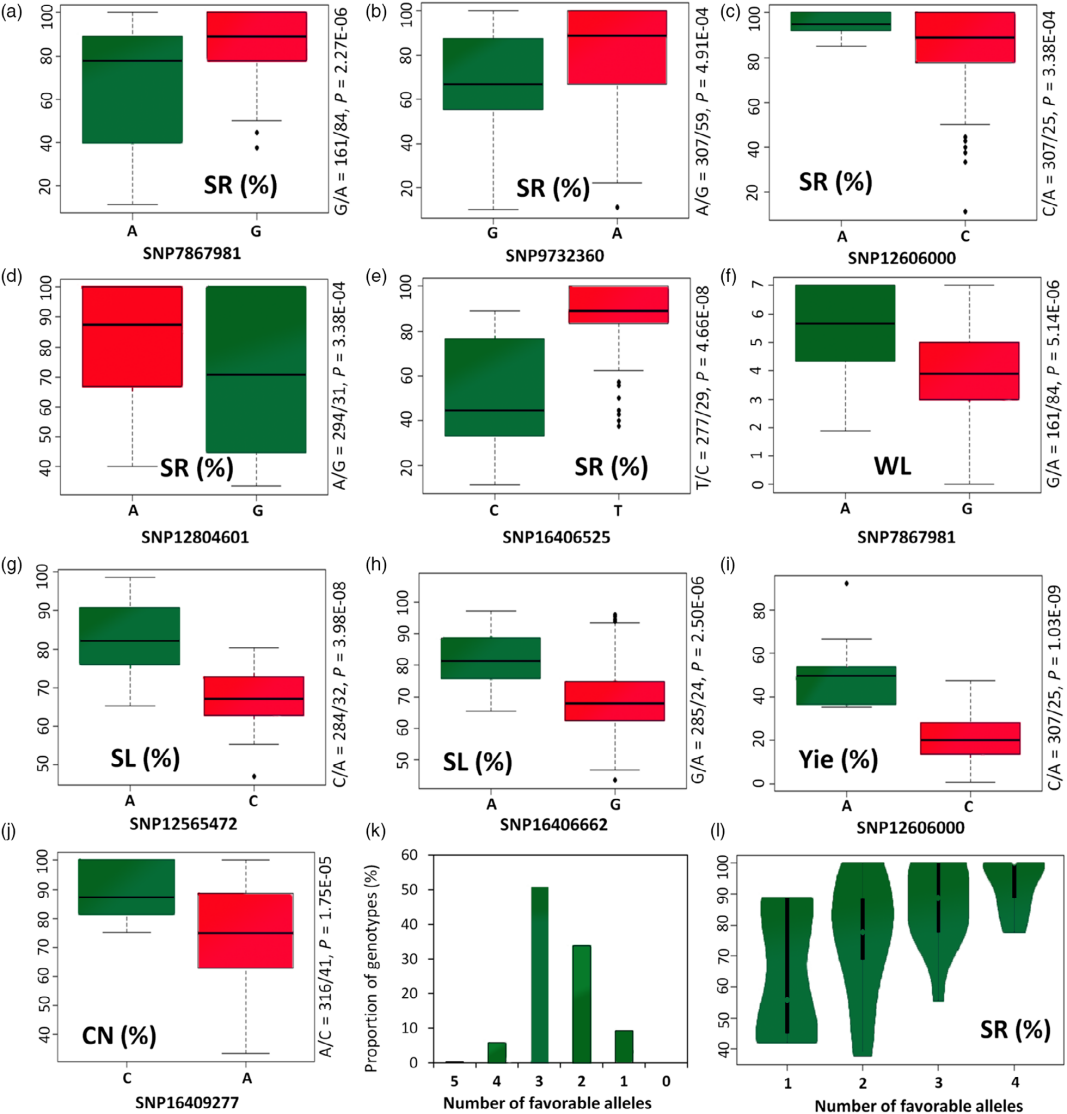
Favourable alleles of the peak loci and their pyramiding effects on drought tolerance in *Sesamum indicum*. (a) Effects of alleles G/A of the locus SNP7867981. (b) A/G at the locus SNP9732360. (c) C/A of the locus SNP12606000; (d) A/G at the locus SNP12804601. (e) T/C at the locus SNP16406525 on plant survival rate (SR); (f) Effects of alleles G/A of the locus SNP7867981 on the wilting level (WL). (g) Effects of alleles C/A of the locus SNP12565472. (h) G/A of the locus SNP16406662 on the relative stem length (SL). (i) Effects of alleles C/A of the locus SNP12606000 on the relative seed yield (Yie). (j) Effects of alleles A/C of the locus SNP16409277 on the relative capsule number (CN). Data are from the year 2016 experiment and the trait values of the two haplotype groups were compared using *t*‐tests; the green and red boxes represent the variant allele and common allele, respectively. (k) Proportion of sesame accessions harbouring combination of favourable alleles at the peak loci. (l) Pyramiding of favourable alleles at the loci SNP12606000, SNP9732360, SNP16406525 and SNP7867981 improves the survival rate (SR) of sesame accessions.

We defined at each peak, the alleles that led to the increase in SR, relative Yie, CN and SL indexes and decrease in WL as the favourable ones. Concerning the SR, amongst the five associated peak loci, the desirable alleles were found to be mainly the common alleles contributing to the enhancement of sesame plant survival under drought stress (Figure 3a–e). Similarly, the common allele G at the locus SNP7867981 lowered the wilting signs of the plants under drought stress (Figure 3f). For the locus SNP12565472 tightly linked to the SL index, the variant allele A significantly increased drought tolerance compared with the common allele C (*P* < 0.001). The variant allele A at the second locus SNP16406662 associated with SL index, also significantly increased drought tolerance compared to the common allele G (*P* < 0.001) (Figure 3g,h). A similar result was observed for the relative seed yield (Yie) index which was strongly improved in the accessions harbouring the variant allele A at the locus SNP12606000 (*P* < 0.001) under drought stress condition (Figure 3i). Finally, the variant allele C at the locus SNP16409277 detected for the CN index, significantly enhanced capsule formation under drought stress condition as compared to the common allele (*P* < 0.001) (Figure 3j). Taken together, our results showed that alleles which are crucial for the plant survival under drought stress conditions have already been fixed in the sesame crop and have been positively selected by recent breeding (Table S4). However, alleles that are essential for productivity and yield maintenance under drought conditions have not yet been intensively selected and are far from being fixed in most of the accessions. In the association panel, the majority of the accessions (50.75%) displayed a combination of three desirable alleles (Figure 3k). Furthermore, the accessions from the tropical area (coloured in green) had more drought‐tolerance alleles at the peaks than those from the northern area (coloured in red), which may underpin their higher drought tolerance (Figure S5A).

To determine whether the favourable alleles at the peaks have a positive pyramiding effect, we selected the four strongest associated loci (SNP12606000, SNP9732360, SNP16406525 and SNP7867981) and analyzed the SR and relative Yie data. The analysis revealed that the accumulation of favourable alleles contributes to higher SR and Yie maintenance under drought stress condition (Figure 3l; Figure S5B). Therefore, future breeding efforts may focus on pyramiding as much as possible the desirable alleles at the peak loci into elite sesame cultivars.

### Detecting the candidate genes for drought tolerance in sesame

**Figure 4:**
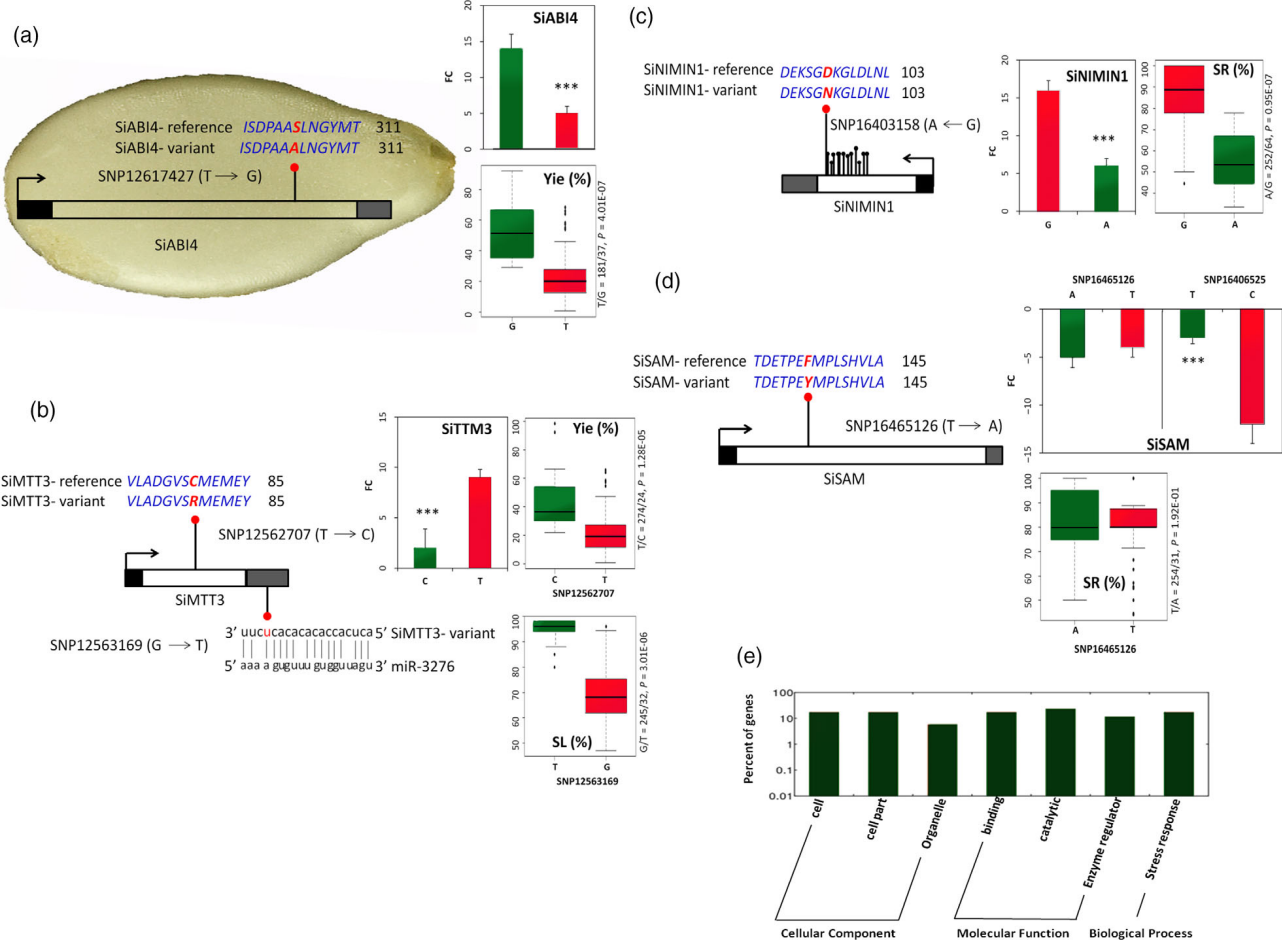
Detecting some candidate genes containing significant SNPs in the stable and pleiotropic drought tolerance QTLs in *Sesamum indicum*. (a) A missense mutation at the locus SNP12617427 in the coding region of the seed‐specific gene *SiABI4* alters the gene expression and the relative seed yield (Yie) between the two haplotype groups; FC means expression fold change and the trait values of the two haplotype groups were compared using *t*‐tests. Error bars indicate the SD of biological replicates, (***P < 0.001). (b) Two missense mutations in the coding region and UTR‐3′ significantly affects the expression of *SiTTM3* and drought tolerance. The polymorphism at the locus SNP12562707 alter the relative seed yield (Yie) between the two haplotype groups while the variant at the locus SNP12563169 influences the binding of miR‐3276 to *SiTTM3* and significantly affects the relative stem length (SL). (c) Several synonymous SNPs located in the coding region and a strongly associated missense change at the locus SNP16403158 of the gene *SiNIMIN1* significantly affects the gene expression level and the survival rate (SR) between the two haplotype groups. (d) A missense mutation in the coding region of *SiSAM* (SNP16465126) did not significantly alter the gene expression and the survival rate (SR) between the 2 haplotype groups. However, *SiSAM* expression was significantly different between the 2 haplotype groups at the peak locus SNP16406525 of the QTLSR8.1, suggesting the existence of another causal variant in *SiSAM*. (e) Gene ontology analysis of the potential candidate genes around the peaks selected based on their expression fold change under drought treatment.

We used the genome information of sesame to uncover the candidate genes associated with each peak signal. All genes in the LD region surrounding the peaks (both single‐year and multi‐year peaks) were determined (Table S5). Based on the ten stable QTLs, we identified a total of 102 genes ranging from 12 to 30 genes around each peak (Table 2). In many cases, the peaks were not located in genic or regulatory regions, but co‐segregating significant SNPs were found directly in genic regions. These gene‐containing significant SNPs have high potentials to modulate drought tolerance in sesame (Table S6). The gene *SIN_1012139* (*SiABI4*, ABA insensitive 4) from the QtlY4.1 contains a missense mutation at the locus SNP12617427 (T/G, MAF = 5%) located in the coding region, which changes the amino acid S to A (at the 305th position). Accessions with the variant allele G have higher relative seed yield index than those with the common allele T (Figure 4a). *SiABI4* was not expressed in leaf, stem and even root tissues of both T allele‐accessions and G allele‐accessions. However, it was highly induced under drought stress in the seed of G allele‐accessions and slightly in T allele‐accessions. Our results denote that *SiABI4* is a seed‐preferential gene and the mutated allele G enhances sesame seed yield under drought conditions. Another gene containing a missense mutation detected in this QTL is *SIN_1012134* (*SiTTM3*, Triphosphate tunnel metalloenzyme 3). The polymorphism at the locus SNP12562707 (T/C, MAF = 5%) located in the coding sequence changes C to R at the 80th amino acid. Accessions with the mutation have more than the double of the relative seed yield index as compared to those with the T allele, which is the perfect opposite at the gene expression levels in leaf tissues (Figure 4b). These results indicate that *SiTTM3* is probably a negative modulator of drought tolerance in sesame, a function which has not yet been reported for its homolog in *Arabidopsis* (*AtTTM3*). The same gene (*SiTTM3*) was found associated with SL, but another missense change in this gene at the locus SNP12563169 (G/T, MAF = 5%) located in the 3′‐UTR region likely affects SL index (Figure 4b). Accessions with the variant allele displayed a strong tolerance to drought as shown by their high SL index value. *In silico* analysis in miRBase (www.mirbase.org) of potential microRNA binding sites in the 3′‐UTR region of *SiTTM3* revealed that the T variant leads to the gain of a putative binding site for miR‐3276 which cannot bind *SiTTM3* harbouring the G allele (*E*‐value cut off = 4). Although there is no perfect seed matching, the T variant strengthens a potential compensatory base pairing site in the 3′‐region of the miR‐3276 (Figure 4b). We inferred that the miR‐3276 binds to the 3′‐UTR region of the T‐allele *SiTTM3* and inhibits its expression posttranscriptionally. Interestingly, the two SNPs located in *SiTTM3* (SNP12562707 and SNP12563169) were in a strong LD (*r* ^2^ = 0.9), therefore, both loci may cooperate to enhance yield and productivity maintenance under drought conditions in sesame.

In the QtlSR8.1, the gene *SIN_1022782* (*SiNIMIN1*, NIM1‐Interacting 1), contained 44 significant SNPs and most of them were synonymous mutations. However, the peak locus SNP16403158 (*P* = 2 × 10^−7^, MAF = 4%) is a missense change (G/A) that led to D/N variation at the 96th amino acid of the SiNIMIN1 protein. Phenotypic analysis of the alleles at the locus SNP16403158 showed that the variant allele strongly decreased the survival rate under drought stress of sesame plants. Moreover, *SiNIMIN1* was found weakly expressed in leaves of accessions carrying the variant allele than the G‐allele accessions (Figure 4c). The homolog of *SiNIMIN1* in *Arabidopsis* (*AtNIMIN1*) has been described to be involved in plant defence response, therefore, its implication in drought tolerance in sesame suggests a functional diversification. In the same QTL region, another important gene *SiSAM* (*SIN_1022789*,* S*‐adenosylmethionine synthetase) containing a missense significantly associated SNP (SNP16465126, MAF = 5%) was uncovered. The mutation affects the F amino acid at the 137th position which changes to Y. *SiSAM* was highly down‐regulated in sesame leaves under drought stress but we did not observe an obvious difference between accessions carrying the common allele T and the variant A at the locus SNP16465126 (*P* > 0.05). Similarly, both alleles did not differentially affect the survival rate of sesame plants under drought stress (Figure 4d). However, *SiSAM* gene expression was significantly different when we compared accessions harbouring the T and C allele at the peak locus SNP16406525 of this QTL (QtlSR8.1). These observations suggest the existence of another causative variant which alters *SiSAM* expression and has been probably excluded from the genotyping data due to missing data. *SiSAM* homologous gene (*AtSAM1*) in *Arabidopsis* is well described as a stress responsive gene, therefore, over‐expression of *SiSAM* harbouring the favourable allele may represent an efficient way for drought tolerance improvement in sesame.

Other candidate genes‐containing significant SNPs such as *SIN_1015691* (*SiP450*), *SIN_1015693* and *SIN_1004723* were detected in the remaining stable QTLs and may be valuable genetic resources for improving drought tolerance in sesame (Table 2). With regard to the QTLs for which we did not find any candidate gene‐containing SNPs, we referred to the functional annotation of all genes located within the QTL and also used drought stress transcriptome datasets developed in our group (available at the NCBI sequence read archive under accession numbers: SAMN09517829 and SAMN09517828). This approach helped identify some potential candidate genes including *SIN_1022774* (*SiGOLS1*), *SIN_1005662* (*SiPOD3*) and *SIN_1004716* (Table 2, Table S7). Gene ontology analysis of all candidate genes detected in this study indicated that they are involved in the biological process related to stress responses and play molecular functions related to catalytic, binding and enzyme regulator activity (Figure 4e).

### A missense change in the coding region of *SiSAM* modulates drought tolerance in sesame

*S*‐adenosylmethionine synthetase genes have been reported to be involved in abiotic stress responses in various plant species (Kim *et al*., 2015; Lin *et al*., 2008; Radadiya *et al*., 2016; Wang *et al*., 2016a;; Wang *et al*., 2016b). In this study, *SiSAM* was identified in a major hub locus of the drought tolerance related association network (Figure 2c) but the causative genetic variant in *SiSAM* altering drought tolerance has yet to be discovered. In this regard, we conducted an in‐depth investigation of the natural variation in this gene and also explored its implication in drought tolerance using yeast and *Arabidopsis thaliana* as models, since sesame genetic transformation is not yet very effective. *SiSAM* is an intronless gene with a transcript length of 1182 bp and 393 amino acid protein length. *SiSAM* promoter contains several abiotic stress and hormonal cis‐acting regulatory elements, particularly the ABRE motif, suggesting that *SiSAM* activity is likely to be ABA‐dependent (Figure S6).

**Figure 5:**
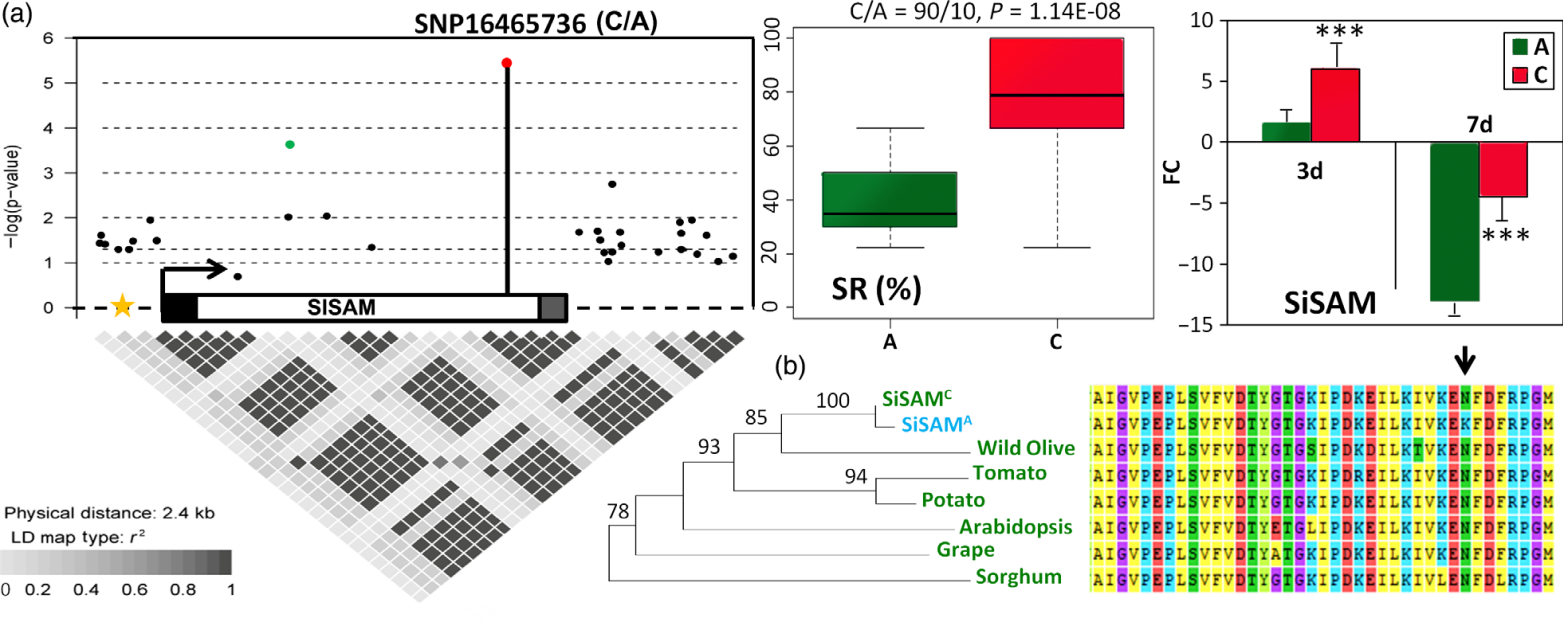
Candidate gene association analysis of *SiSAM*, a drought tolerance candidate gene in *Sesamum indicum*. (a) Local manhattan plot and LD statistic *r* ^2^ for *SiSAM* (gene body, 400 bp upstream and 1.2 kb downstream of the gene). The white, black and gray rectangles represent the exon, UTR‐5′ and UTR‐3′, respectively. The arrow indicates the transcription start site and transcription orientation. The star represents the position of the ABRE cis‐acting regulatory element in the promoter region of *SiSAM*. The black dots represent the non‐associated SNPs and InDels whereas the green and red dots indicate the significantly associated SNPs at the loci SNP16465126 and SNP16465736, respectively. The novel and strongest associated loci SNP16465736 significantly affects the survival rate (SR) and gene expression fold change (FC) at 3 and 7 days under drought stress in the two haplotype groups. Error bars indicate the SD of biological replicates, (***P < 0.001). (b) The homologs of *SiSAM* in plants and local alignment of protein sequences of the homologs around the candidate causative variant SNP16465736 (indicated by the arrow).

In order to fully identify the DNA polymorphism present in the gene *SiSAM*, it was re‐sequenced in 100 randomly selected sesame accessions (Table S8). A 2.4 kb genomic fragment was sequenced spanning the *SiSAM* coding region, both the 5‐, and 3‐untranslated regions (UTR) and the promoter. In total, 28 SNPs and 5 insertions‐deletions were discovered (MAF ≥ 0.05). These polymorphisms included the locus SNP16465126 already detected in the QtlSR8.1 from the GWAS analysis. We analyzed the association of each polymorphism site with SR using the Mixed linear model and the pairwise LD of the polymorphisms was calculated (Figure 5a). From the 33 polymorphism sites, only two loci, SNP16465736 and SNP16465126, were significantly associated with SR variation (−logP = 5.41 and 3.67, respectively). The novel identified locus SNP16465736 (C/A) is located in the coding region of *SiSAM* and is a missense change leading to N/K variation at the 340th amino acid of the protein. Moreover, the locus SNP16465736 together with the previously identified genic loci SNP16465126 and the peak loci SNP16406525 at the QtlSR8.1, were in LD (*r* ^2^ = 0.8, 0.7, respectively) amongst the sequenced accessions. The common allele C at the locus SNP16465736 contributes to a higher SR than the variant allele. Similarly, the gene *SiSAM* was strongly up‐regulated as an early response to drought stress, but higher in the C‐allele accessions than in the A‐allele accessions. Subsequently, the transcript level of *SiSAM* was significantly more decreased in the accessions harbouring the variant allele A as compared to those with the C allele under prolonged drought stress (Figure 5a). These results indicate that the polymorphism at the locus SNP16465736 is likely to be the causative variant altering the expression and function of *SiSAM* under drought stress in sesame. The predicted drought tolerant allele (encoding the amino acid N) is the ancestral type according to the phylogenetic analysis with its homologs in other plants (Figure 5b) and information from wild *Sesamum* species. Interestingly, we found that all accessions with the A allele are from the northern area group, which accessions were found to be more sensitive to drought stress (Figure 1e). Sesame is thought to have originated in Africa or South Asia (Bedigian, 2003; Hiltebrandt, 1932) and the northern area group may be derived from the long‐term selection for adaptation to the photoperiod change (Wei *et al*., 2015). We therefore speculate that the mutated A allele may have facilitated sesame acclimation to the northern area.

**Figure 6:**
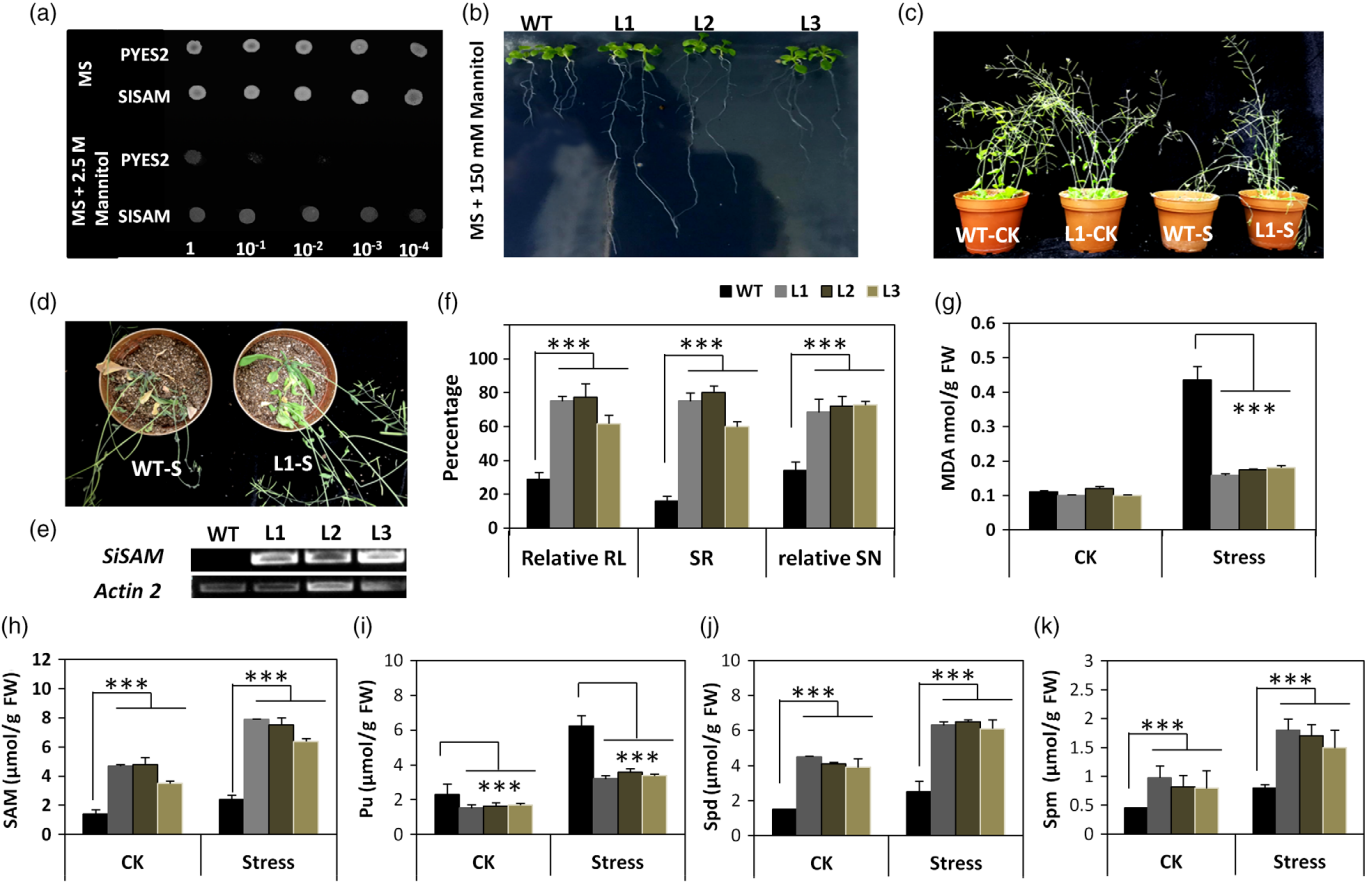
Functional analysis of *SiSAM*, a drought tolerance candidate gene in *Sesamum indicum*. (a) Various serial dilutions of yeast transformants carrying the candidate gene *SiSAM* ^*C*^ could grow normally in a MS medium supplemented with 2.5 m Mannitol in contrast to the yeast transformed with the control vector PYES2. (b) Phenotypes of wild type (WT) and transgenic *Arabidopsis thaliana* plants (L1‐L3) over‐expressing *SiSAM* ^*C*^, under 150 mm Mannitol after 7 days. (c–d) Phenotypes of wild type (WT) and transgenic *Arabidopsis thaliana* plants (L1) over‐expressing *SiSAM* ^*C*^, after 17 days water stress (S) and normal (CK) conditions. (e) RT‐PCR analysis of transcript levels of *SiSAM* ^*C*^ in the three transgenic lines and wild type (WT) plants. (f) Relative root length (RL), survival rate (SR) and relative silique number (SN) of the three transgenic lines and wild type (WT) plants. (g) Malonaldehyde (MDA) content of the three transgenic lines and wild type (WT) plants after 17 days water stress (S) and normal conditions (CK); (H‐K) Total adenosyl methionine (SAM), putresine (Put), spermidine (Spd) and spermine (Spm) in wild type (WT) plants and transgenic *Arabidopsis thaliana* lines (L1, L2 and L3) over‐expressing *SiSAM* ^*C*^ after 17 days water stress (S) and normal (CK) conditions. The average data and standard errors were calculated from three independent experiments. Bars with asterisks indicate lines that are significantly different to the wild type plants (***P < 0.001).

We transferred *SiSAM* containing the C allele (named *SiSAM* ^*C*^) allele into yeast and *Arabidopsis* to further validate its implication in stress tolerance. We observed that the yeast transformants carrying *SiSAM* ^*C*^ could grow normally in osmotic stress‐imposed medium containing 2.5 m Mannitol while the transformed yeasts with the empty plasmid could not restore growth at lower dilutions (Figure 6a). Osmotic stress always occurs simultaneously with drought and its tolerance is a vital part of drought tolerance (Farooq *et al*., 2009). We deduced that *SiSAM* ^*C*^ improves osmotic stress tolerance in yeast.

In *Arabidopsis thaliana*, the studied three independent transgenic lines over‐expressing the *SiSAM* ^*C*^ gene all exhibited significantly enhanced drought tolerance than the wild type (WT). Transgenic plants especially the lines L1 and L2 grew better than WT in the osmotic stress‐imposed medium containing 150 mm Mannitol (Figure 6b). Furthermore, after 17 days drought stress treatment, the WT plants were severely wilted, whereas *SiSAM* ^*C*^ over‐expressing transgenic lines showed less wilting signs, thus a stronger drought tolerance than the WT plants (Figure 6c,d, Figure S7). The survival rate of the WT plants was 16%, while the survival rate of the *SiSAM* ^*C*^ over‐expressing lines (Figure 6e) ranged from 60% to 80% (Figure 6f). Similarly, the silique formation was significantly impaired in the WT plants as compared to the transgenic lines under drought stress treatment (*P* < 0.001) (Figure 6g). Transgenic lines have a higher ability for ROS detoxification than the WT plants as shown by the significant lower contents of malonaldehyde (*P* < 0.001), which could explain the low plant wilting signs and the high survival rate (Figure 6g). An analysis of the polyamine spectrum and SAM content indicated that drought stress induced accumulation of putrescine (Put), spermidine (Spd), spermine (Spm) and SAM in WT and transgenic lines (Figure 6h–k). However, we observed significantly higher contents (~3‐fold) of SAM, Spd and Spm in the transgenic lines than in the WT plants under both control and drought stress conditions (*P* < 0.001). Conversely, Put was significantly higher in the WT plants than in the transgenic lines under control and stress conditions (*P* < 0.001). Intriguingly, the gene *AtADC1* involved in Put biosynthesis showed higher transcript level in the transgenic plants than in the WT plants (Figure S8). Also, we noticed that the activities of the transgene and key polyamine metabolism and biosynthetic genes (*AtSAMDC1, AtSAMDC2*,* AtSPDS1*,* AtSPDS2* and *AtSPMS*) were strikingly enhanced in the transgenic lines as compared to the WT plants under drought stress (Figure S8). We deduce from these results that over‐expression of *SiSAM* ^*C*^ in *Arabidopsis* improves SAM accumulation, induces enhanced production and conversion of Put to Spd and Spm, leading to an effective ROS homeostasis and drought tolerance. *SiSAM* ^*C*^ not only improved the survival of the transgenic *Arabidopsis* plants but also allowed good productivity maintenance under drought conditions, exactly as the QtlSR8.1 in sesame (Figure 2c). Altogether, these data support the premise that natural variation in *SiSAM* alters drought tolerance in sesame accessions. Importantly, the favourable allele of *SiSAM* represents a good marker for the development of drought‐tolerant sesame cultivars using traditional breeding approaches.

## Discussion

Phenotype‐genotype association analysis has become a critical tool for identifying alleles and loci responsible for the agronomically important traits (Wan *et al*., 2017). In the current study, the selection of sesame accessions from diverse origins, with sufficient genetic variation and weak population structure is advantageous for GWAS implementation (Cao *et al*., 2016; Wei *et al*., 2015). The LD decay rate in sesame, a self‐pollinated crop (∼88 kb) is relatively lower than those of rice (∼100 kb) (Huang and Han, 2014), sorghum (∼150 kb) (Morris *et al*., 2012) and soybean (∼150 kb) (Lam *et al*., 2010) but higher than the LD decay rate of outcrossing species such as maize (∼2 kb) (Li *et al*., 2013). The modest rate of LD decay in sesame suggests that the resolution of GWAS may not easily resolve to the causative gene. However, by employing a high marker density (5 SNPs/kb) in the current study, we successfully identified QTL regions harbouring a very limited number of genes and several gene‐containing SNPs. Similar to our results, the previous comprehensive GWAS based on an ample marker density for oil quality and agronomic traits in 705 sesame lines was very efficient with the identification of several causative genes (Wei *et al*., 2015).

We detected a total of 569 significant SNPs with modest *P* values (*P* < 7.8 × 10^−6^) distributed across 10 LGs of the sesame genome. Ten major QTLs constitutively identified in the 2 years could be targeted in breeding programmes with high confidence. Collectively, the peak loci at these major QTLs could explain only 40% of the total phenotypic variation. This is understandable since drought tolerance is as a very complex trait involving dynamic and diverse responses that are controlled by a large number of small‐effect loci (Guo *et al*., 2018). It is worth mentioning that we mainly focused on the stable (multi‐year) QTLs in this study, hence, adding other single‐year QTLs will increase the phenotypic contribution. However, we believe that extending our association panel to a more diverse sample particularly, integrating more accessions from Africa could increase the power to detect novel drought tolerance trait‐associated variants using GWAS (Huang *et al*., 2012). Most of the sesame accessions harboured several favourable alleles at the associated loci. In addition, the group of accessions from the tropical area (more prone to drought) had more favourable alleles and was globally more tolerant to drought compared to the northern area accessions. All these findings indicate that being originated from drought‐prone environments of Africa or India (Bedigian, 2003; Hiltebrandt, 1932) and as a result of a long‐term adaptation, sesame has fixed several drought‐tolerance alleles. Hence, the crop has been genetically shaped to survive drought. Similar conclusions were drawn by Liu *et al*. (2013) who observed that maize varieties from the subpopulation consisting of tropical inbred lines (environment more prone to drought) had the highest frequency of the favourable allele of the drought tolerance gene *ZmDREB2.7*, which was consistent with the observed higher level of drought tolerance of this subpopulation than the others.

Most of the important food crops are sensitive to drought stress (Farooq *et al*., 2009) and very few are able to survive drought like sesame (Langham, 2007). In this study, we discovered that the alleles beneficial to the plant survival under drought stress have been fixed and intensively selected in sesame, which can explain the relatively drought tolerance of this crop. However, alleles that are essential for high productivity and yield maintenance under drought conditions are far from being fixed and could be the focus in future sesame breeding programmes. To date, many studies have demonstrated that marker‐based gene pyramiding is very effective in crop improvement strategies (Barloy *et al*., 2007; Sacco *et al*., 2013; Werner *et al*., 2005; Zhang *et al*., 2014). The positive pyramiding effects of the favourable alleles uncovered in our report may be of high potential for the development of drought tolerant sesame cultivars with enhanced capacity for productivity and yield maintenance under stress conditions using allele‐specific molecular markers (Bang *et al*., 2007). In fact, the marker‐assisted breeding approach will be more practical for improving sesame drought tolerance than the transgenic approach since sesame genetic transformation is still at the early stages (Chowdhury *et al*., 2017).

Various novel genes have been reported in this study as involved in sesame drought tolerance. For example, the homolog of the gene *SiNIMIN1* in *Arabidopsis* (*AtNIMIN1*) was described as a pathogen defence responsive gene (Zwicker *et al*., 2007) and a function in abiotic stress have not yet been reported for this gene. Similarly, for the gene *SiTTM3* which likely negatively modulates drought tolerance in sesame (Figure 4), its homolog in *Arabidopsis* (*AtTTM3*) has been shown to play a role in root development (Moeder *et al*., 2013) but no evidence of its implication in drought stress was reported. The gene *SIN_1004716* is the homolog of *AT5G16010* in *Arabidopsis*, which contributes to wax biosynthesis. Plant epidermal wax forms a hydrophobic layer covering aerial plant organs which constitutes a barrier against uncontrolled water loss and biotic stresses (Costaglioli *et al*., 2005). By detecting this gene associated with WL and SR, we inferred that it may be involved in wax accumulation in sesame tissues delaying wilting signs and death of sesame plants under drought stress. Although our genetic analysis identified these genes as drought tolerance candidate genes in sesame, their functional mechanisms have to be elucidated.

Our study also uncovered some known drought tolerance genes previously reported in other plant species. The gene *ABI4* is an example of drought tolerance gene reported in *Arabidopsis* (*AtABI4*) which is involved in abscisic acid signal transduction. Interestingly, *AtABI4* is more active in developing siliques and seed, exactly as the preferential expression of *SiABI4* in sesame seeds, indicating a functional conservation of this gene in the two species (Finkelstein *et al*., 1998). Another important gene uncovered in our study is the gene *SiSAM*, the homolog of *AT1G02500* (*AtSAM*) in *Arabidopsis*. S‐Adenosylmethionine (*SAM*) is required for the biosynthesis of phenylpropanoid compounds and is also a precursor for the biosynthesis of the phytohormone ethylene and polyamines (Kende, 1993; Tiburcio *et al*., 1990). Polyamines are protective molecules and play vital roles in the regulation of plant tolerance to abiotic stress through direct interactions with other metabolic routes and hormonal cross‐talk, and the activation of the expression of stress‐responsive genes (Alcázar *et al*., 2010; Pál *et al*., 2015). *SAM* was reported to modulate polyamine levels and improve tolerance to various abiotic stresses including drought in *Arabidopsis* (Kim *et al*., 2015), broomcorn millet (Lin *et al*., 2008), tomato (Gong *et al*., 2014), soybean (Wang *et al*., 2016a,b) and pigeon pea (Radadiya *et al*., 2016). In this study, we found that a functional variation at the locus SNP16465736 in the coding region of *SiSAM* contributes to the natural variation of drought tolerance in sesame. Further functional investigations showed that *SiSAM* ^*C*^ modulates the polyamine levels, leading to enhanced ROS homeostasis and drought tolerance. However, how the A allele (*SiSAM* ^*A*^) reduces drought tolerance and its likely implication in sesame acclimation to the northern area remains unclear and will need further investigations.

In summary, we revealed for the first time, the allelic variations modulating drought response in sesame and provided genetic evidences explaining how sesame is intrinsically a relatively drought tolerant crop. We further discovered the highly anticipated genes associated with these genetic variants which will fuel future research on molecular improvement of sesame varieties and related crops for higher performance in drought‐prone environments.

## Methods

### Plant materials and phenotyping procedure

A collection of 400 sesame (*Sesamum indicum* L.) accessions, including modern cultivars and landraces was used for the GWAS in this study. These accessions were extracted from a large collection of more than 8000 sesame accessions preserved at the China National Gene Bank, Oil Crops Research Institute, Chinese Academy of Agricultural Sciences. These samples were selected because they have a similar flowering period (Wei *et al*., 2015) and originated from 29 different countries all over the world (Table S2). Unfortunately, accessions from Africa were less represented in our panel mainly because most of these materials could not grow well in the phenotyping environment.

The 400 accessions were self‐pollinated for four generations in Sanya, Hainan province, China (109.187°E, 18.38°N, altitude 11 m). For this experiment, the germplasm were grown in pot (25 cm diameter and 30 cm depth) containing 7 kg of loam soil with known physicochemical properties mixed with 10% of added compound fertilizer. The pots were arranged in a greenhouse at OCRI‐CAAS's research station in Wuhan, Hubei Province, China (114.31°E, 30.52°N, altitude 27 m). The experiment was carried out under a completely randomized split‐plot from May to September in 2015 and 2016. The 400 accessions and water regimes (control and drought stress) were arranged as sub‐plot and main‐plot, respectively. With two plants per pot and three replicated pots for the same genotype in each treatment (water regime), we obtained a total of 7200 plants for phenotyping. Seedlings were well‐watered to keep the maximum soil moisture condition (100% field capacity [FC]) and ensure plants’ normal growth. Drought stress was applied when 90% plants entered early flowering stage and spanned the reproductive phase at which sesame is the most sensitive to drought stress (Sun *et al*., 2010). Water was withheld in the drought treatment for 7 days (~30% FC). The 8th day from the beginning of the drought stress application, the stressed plants were watered to reach 100% FC to help recovering. The drought stress application and rewatering scheme were further carried on two more times, and afterwards, the irrigation was kept normally until the maturation stage. Meanwhile, the control plants were normally irrigated during the whole experiment. The variations of the mean temperature and relative humidity during the repeated stress periods are presented in Figure S9.

### Phenotypic evaluation of the association panel

Five drought related traits were investigated including the wilting level of the whole plant (WL) recorded on the stressed plants at the end of the last drought stress treatment before rewatering; the survival rate (SR) evaluated on the stressed plants a week after the last drought stress treatment after rewatering; the stem length (SL), capsule number per plant (CN) and seed yield (Yie) at the maturation stage on all plants (control and stress). A scale of 0–7 was used for visual scoring of WL with 0 meaning no wilting sign and 7 for completely wilted or dead plants. SL (cm) was measured as the length from the base of the stem to the tip of the main inflorescence. CN is the total number of capsules per plant and Yie (g) is the weight of all seeds produced per accession. Yie was recorded only in 2016. During the 2 years, the phenotyping procedure and scoring standard were the same.

### Statistical analysis

All statistical analyses were performed with the R package (R Core Team, 2015). Least square means were calculated with general linear model procedure for the replications of each accession in the respective years and for both control and drought stressed plants. Drought tolerance index was estimated for SL, CN and Yie according to Long *et al*. (2013) using the formula: 

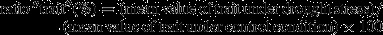




In addition, broad‐sense heritability (*H* ^2^) across the treatments was calculated as follow:

$$H^{2}=\sigma_{a}^{2}/\left(\sigma_{a}^{2}+\sigma_{\mathrm{ay}}^{2}/Y+\sigma_{\varepsilon }^{2}/\text{YR}\right)$$

Where: $\sigma_{a}^{2}$, $\sigma_{\mathrm{ay}}^{2}$ and $\sigma_{\varepsilon }^{2}$ are estimates of the variances of accession, accession × year interaction and error, respectively. *Y* represents year, and *R* is the number of replications (Kowles, 2001).

ANOVA was performed using the packages ‘Ade4’ (Dray *et al*., 2007) and ‘Agricolae’ (de Mendiburu, 2014). The normal distribution of data was determined using the Shapiro–Wilk *W*‐test, whereas the homogeneity of variances was determined with the Bartlett test. Differences were tested for significance at the 5% probability level and mean comparisons were done using the Tukey HSD test.

### Population genetics and GWAS

A total of 1 000 939 common SNPs covering the whole genome with minor allele frequency (MAF) >0.03 were obtained from the sesame HapMap project (www.ncgr.ac.cn/SesameHapMap/) and used for trait‐association analysis in this study (Wei *et al*., 2015). The quality control and linkage disequilibrium (LD) estimation were performed as described previously (Wei *et al*., 2015). The software MEGA7 (Kumar *et al*., 2016) was employed to construct a phylogenetic tree based on simple matching coefficients. Principal component analysis of the population was performed in the R package (R Development Core Team, 2015). Additionally, the program STRUCTURE 2.3.4 (Falush *et al*., 2003) was used for a bayesian clustering analysis with the admixture model and correlated allele frequencies. Five runs were performed for each subpopulation K (1–10) (Dossa *et al*., 2016). The burn‐in time and iterations for each run were set to 30 000 and 70 000 and the true *K* was determined according to the method described by Evanno *et al*. (2005). All accessions were mapped according to their geographical coordinates using the Tableau software version 9.3 (Seattle, WA). The relative kinship analysis was implemented using the software package SPAGeDi (Hardy and Vekemans, 2002). Association analysis was performed using the EMMAX package (Kang *et al*., 2010) based on the Mixed model and the matrix of pairwise genetic distance derived from simple matching coefficients was used as the variance–covariance matrix of the random effect.

Suggestive (1/*n*) *P*‐value threshold was set to control the genome‐wide type 1 error rate derived from the Mixed model (Duggal *et al*., 2008); *n* represents the effective number of independent SNPs calculated using the Genetic Type I Error Calculator software (Li *et al*., 2012). The estimated effective number of independent tests was 128 405 SNPs and the threshold used to identify significantly associated loci was set at *P* = 7.8 × 10^−6^. The Manhattan, regional and QQ plots were generated using the EMMAX and the R packages, qqman (Turner, 2014), snp.plotter (Luna and Nicodemus, 2007). The association analysis was performed independently on single‐year phenotypic datasets and the repeated significant signals (over years) were considered as the most reliable. For clustered significant SNPs, the SNP with the highest −log_10_ (P) in the LD region were considered as the peak. Significant associations were also selected on the threshold of *P* ≤ 0.01, corrected for multiple comparisons according to the false discovery rate procedure reported by Benjamini and Hochberg (1995). Regions of 88 kb (corresponding to the LD window) upstream and downstream of the peaks and harbouring at least two clustered significant loci, were defined as quantitative trait loci (QTL). The value *r* ^2^ derived from linear regressions were calculated to examine the phenotypic variance explained (PVE) of each peak in the R package (R Development Core Team, 2015). Before fitting the model, each marker was coded with the value 0 used for the reference allele and the value 1 for the alternative allele. Next, all genes were extracted from the genomic regions with two adjacent windows of LD centred by a peak loci to seek for the candidate genes. The corresponding putative homologs of all candidate genes in *Arabidopsis thaliana* were obtained from the database SesameFG (http://ncgr.ac.cn/SesameFG/; Wei *et al*., 2017). Gene ontology annotation of these genes was carried out using Blast2GO tool v.3.1.3 (Götz *et al*., 2008) and plotted with the WEGO tool (Ye *et al*., 2006).

### Candidate gene association study

The candidate gene *SiSAM* (*SIN_1022789*) located in a pleiotropic QTL significantly associated with various drought tolerance indexes was used for an in‐depth analysis. Promoter analysis of *SiSAM* was performed using the online analysis software PlantCARE (Lescot *et al*., 2002). The region located 1 kb upstream of *SiSAM* coding sequences was used as the promoter sequence for analyzing cis‐elements. A subset of 100 materials (Table S8) was randomly selected from the panel for candidate gene association study. The *SiSAM* promoter region (400 bp), coding sequences and 3‐untranslated region (UTR) (1034 bp) were isolated by PCR on genomic DNAs obtained from the panel of genotypes using two primers (SiSAM‐1F: 5′‐AGCAGTTTCCTTTAGTCC‐3′, SiSAM‐1R: 5′‐ACCAGAGGGATTGAGGT‐3′; SiSAM‐2F1: 5′‐CCGTTGAGTACTACAATGAAA‐3′, SiSAM‐2F2: 5′‐GCATACGGTCACTTTGGAAGAGAC‐3′, SiSAM‐2R: 5′‐ACATGTACCGAATTCTAAAAGAGCTAG‐3′) designed according to the *SiSAM* sequence from the sesame reference genome (Wang *et al*., 2014). All the obtained sequences were assembled with the DNAMAN software (Lynnon Biosoft, QC, Canada) and aligned using MEGA version 7.0.20 (Kumar *et al*., 2016). Nucleotide polymorphism including SNPs and Insertion‐Deletion (MAF ≥ 0.05) were identified with the DNASP software v6.11.01 (Rozas *et al*., 2017).

Genotypic and phenotypic data files were prepared and imported to TASSEL5.0 (Bradbury *et al*., 2007) for association analysis. The Mixed linear model (MLM), taking account of both the kinship coefficients and the population structure (PCA+K) containing the loci tested as a fixed effect, was applied to evaluate the association between SR and the polymorphisms. MLM is effective to correct for the effect of cryptic relatedness and for controlling false positives in the association analysis (Zhang *et al*., 2010). To get reliable results, *P* ≤ 0.001 was considered to have a significant effect on the trait.

### Expression profiling of candidate genes under drought condition based on qRT‐PCR

Selected accessions showing variation at some significant loci (five accessions for each allele) were submitted to 7 days drought stress at flowering stage and RNAs from the second upper leaves (the seed was used in one case) of stressed and control plants were extracted using the EASYspin Plus kit (Aidlab Biotechnologies, Beijing, China) according to the manufacturer's instructions. The RNA was treated with DNaseI and reverse transcribed with oligo (dT23) primer using the FastQuant RT kit (Tiangen Biotech, Beijing, China). Specific primers for the candidate genes identified were designed by using Primer5.0 (Lalitha, 2000) (Table S9). The qRT‐PCR analyses of the genes were performed using the ChamQ SYBR qPCR Master Mix (Vazyme Biotec, Nanjing, China) on a Light Cycler 480 II (Roche, Basel, Switzerland). The relative expression levels of the genes normalized to the expression level of *actin7* gene (*SIN_1006268*) and *Histone H3.3* gene (*SIN_1004293*) were calculated from cycle threshold values using the $2^{-\Delta \Delta C_{t}}$ method (Livak and Schmittgen, 2001). This analysis was carried out in three independent biological replicates and three technical replicates of each biological replicate.

### Yeast genetic transformation assay

RNA was extracted from 7 days drought‐stressed leaf samples of the accession G049 (harbouring the favourable allele C at the locus SNP16465736 in *SiSAM*) and reversed transcribed as described in the qRT‐PCR experiment. The software Primer5.0 was used to design specific primer pair of the candidate gene *SiSAM* ^*C*^ joined with the primers of the yeast expression vector pYES2 (Invitrogen, Carlsbad, CA) (SiSAMyeastF: 5′‐TACCGAGCTCGGATCCATGGAGACCTTCTTGTTTAC‐3′, SiSAMyeastR: 5′‐GATATCTGCAGAATTCTTAGTTCTGGGGCTTCTCCC‐3′). It was amplified using 2xHigh fidelity Master Mix (MCLAB, Beijing, China) and the yield and quality of the amplicon were monitored on 1% agarose gel. The PCR fragment was excised and purified following Midi Purification Kit protocol (Tiangen Biotech). The purified fragment was ligated into the vector pYES2 according to Exnase II (Vazyme Biotec) kit instructions. DNA from one cloning reaction was used for one transformation of 100 μL *Escherichia coli* Trans1‐T1 Phage Resistant Chemically Competent Cell (Beijing TransGen Biotech Co., Ltd., Beijing, China) and selected on LB agar plates containing 50 μg/mL ampicillin. After 24 h at 37 °C, transformed cells were amplified using NOVA Taq‐Plus PCR Gold Mix (LCP Biomed, Beijing, China) and the positive cells carrying the gene of interest were scraped off from each plate, introduced into 900 mL of LB containing 50 μg/mL ampicillin and shook during 45 min at 200 r.p.m. in a Innova 43 Incubator Shaker Series (New Brunswick Scientific, Edison, NJ). Ten positive clones were fully sequenced (www.tsingke.net) and checked with the reference gene sequence using the BioEdit software (Hall, 1999). About 0.25 μg of plasmid DNA containing the targeted gene was transformed into yeast (*Saccharomyces cerevisiae*) strain INVSc1. Recombinant yeast cells were propagated in the SC‐U liquid medium for 72 h. Cell density was adjusted to OD_600_ at 1.0 followed by serial dilutions 1:10. Ten microliters of each dilution (10^−1^, 10^−2^, 10^−3^ and 10^−4^) were drop plated on SC‐U containing 2.5 m Mannitol, identified as the optimum concentration to impose the osmotic stress. Cultures were incubated for 48 h at 30 °C before photographing.

### Vector construction and *Arabidopsis* genetic transformation

To functionally characterize the gene *SiSAM* (*SIN_1022789*) containing the favourable allele C (*SiSAM* ^*C*^) in *Arabidopsis*, we cloned the protein coding region of *SiSAM* ^*C*^ by PCR from the drought tolerant genotype G049, and inserted the sequence into a pCAMBIA 1301 vector. *Arabidopsis* transformation was carried out using the floral dip method (Clough and Bent, 1998) with *Agrobacterium tumefaciens* strain LBA4404. Transgenic seeds were screened by sowing on MS medium containing 50 μg.ml^‐1^ hygromicin. The T3 transgenic homozygous lines were used for the gene expression assay and phenotypic analysis.

### *Arabidopsis* growth conditions and phenotyping

*Arabidopsis thaliana* ecotype (Col‐0) cv. Columbia was used in this study. The seeds were surface sterilized and plated on MS medium with 2% sucrose and 2 g/L agar. The seeds were stratified for 2 days in the dark at 4 °C and then transferred to growth room at 22 °C under long‐day conditions (16 h light/8 h dark). After a week, transgenic *SiSAM* ^*C*^ seedlings and the wild type were transferred into MS medium supplemented with 150 mm Mannitol to simulate osmotic stress. Seedling root length was recorded after a week. Meanwhile, 15‐day‐old seedlings were transferred to soil and grown in normal conditions for 15 days. Then, half of the plants were subjected to dehydration stress for 17 days during the reproductive phase. The plant survival rate, relative silique number and malonaldehyde content were investigated. Polyamines and SAM contents were investigated as described by Gong *et al*. (2014). The qRT‐PCR was performed a described above using the *Arabidopsis* gene *Actin 2* (*AT3G18780*) as the internal control. Data are presented as relative transcript level ($2^{-\Delta C_{t}}$ ) (Table S9).

## Conflict of interest

They declare no conflict of interest.

## Author contributions

K.D., D.L., L.W. and Y.Z., performed the phenotyping and GWAS data analysis. J.Y., A.L., R.Z., J.Y., M.A.M., X.W. and D.F. assisted in laboratory experiments, data analysis and the manuscript revision. D.D., N.C., X.W. and X.Z. conceived and coordinated the project. K.D. wrote the manuscript. All authors read and approved the final version of the manuscript.

## Funding

This work is financially supported by the China Agriculture Research System (CARS‐14), the Agricultural Science and Technology Innovation Project of Chinese Academy of Agricultural Sciences (CAAS‐ASTIP‐2013‐OCRI), the National Natural Science Foundation of China (31671282, 31500223) and the Fundamental Research Funds for Central Non‐profit Scientific Institution (1610172018007). KD acknowledges the fellowship offered by the Chinese Scholarship Council (2015GXY934).

## Supporting information


**Figure S1** Population genetics of the *Sesamum indicum* association panel.

**Figure S2** Manhattan plots and QQ plots of genome‐wide association studies using the mixed model for relative capsule number (CN) in *Sesamum indicum* during 2015 and 2016.

**Figure S3** Manhattan plots and QQ plots of genome‐wide association studies using the Mixed model for Wiling level (WL) in *Sesamum indicum* during 2015 and 2016.

**Figure S4** Manhattan plots and QQ plots of genome‐wide association studies using the Mixed model for relative seed yield (Yie) trait in *Sesamum indicum* during 2016.

**Figure S5** (A) Boxplot displaying the number of favourable alleles between the two groups (tropical and northern areas) of *Sesamum indicum* accessions; (B) Pyramiding of favourable alleles at the loci SNP12606000, SNP9732360, SNP16406525 and SNP7867981 improves the seed yield maintenance of sesame accessions under drought stress.

**Figure S6** Cis‐acting regulatory elements detected in the promoter of *SiSAM*.

**Figure S7** Phenotypes of wild type (WT) and transgenic *Arabidopsis thaliana* plants (L2‐L3) over‐expressing *SiSAM* ^*C*^, after 17 days water stress (S) and normal conditions (CK).

**Figure S8** Effects of drought stress on relative transcript levels of polyamine metabolism and biosynthesis key genes: *SiSAM* ^*C*^, *AtADC*,* AtSAMDC*,* AtSPDS* and *AtSPMS* in wild type (WT) plants and transgenic *Arabidopsis thaliana* lines (L1, L2 and L3) over‐expressing *SiSAM* ^*C*^.

**Figure S9** Variation of the mean temperature and relative humidity during repeated drought treatment periods on *Sesamum indicum* accessions.

**Table S1** LG wise SNP distribution in *Sesamum indicum*.

**Table S2** Full list of the 400 *Sesamum indicum* accessions used in this study, their origins, breeding status and sequencing information.

**Table S3** Full list of the significant signals identified in 2015 and 2016 for the five traits and their genomic information in *Sesamum indicum*.

**Table S4** Frequency of the favourable alleles at the peak loci in *Sesamum indicum* modern cultivars and landraces.

**Table S5** Full list of the genes in LD region around the peak loci and their functional annotation in *Sesamum indicum*.

**Table S6** List of gene‐containing significant SNPs in the LD region around peak loci detected for *Sesamum indicum* drought tolerance traits in 2015 and 2016.

**Table S7** RNA‐seq based transcript levels of potential candidates genes detected in QTL regions from the GWAS.

**Table S8** List of the 100 accessions used for candidate gene association analysis.

**Table S9** List of the primers used for the qRT‐PCR in *Sesamum indicum* and in *Arabidopsis thaliana*.

## References

[pbi13100-bib-0001] Alcázar, R. , Altabella, T. , Marco, F. , Bortolotti, C. , Reymond, M. , Koncz, C. , Carrasco, P. *et al*. (2010) Polyamines: molecules with regulatory functions in plant abiotic stress tolerance. Planta, 231, 1237–1249. 20221631 10.1007/s00425-010-1130-0

[pbi13100-bib-0002] Anilakumar, K.R. , Pal, A. , Khanum, F. and Bawas, A.S. (2010) Nutritional, medicinal and industrial uses of sesame (*Sesamum indicum* L.) seeds. Agric. Conspec. Sci. 75, 159–168.

[pbi13100-bib-0003] Bahrami, H. , Razmjoo, J. and Jafari, A.O. (2012) Effect of drought stress on germination and seedling growth of sesame cultivars (*Sesamum indicum* L.). Int. J. Agrisci. 2, 423–428.

[pbi13100-bib-0004] Bang, H. , Kim, S. , Leskovar, D. and King, S. (2007) Development of a codominant CAPS marker for allelic selection between canary yellow and red watermelon based on SNP in lycopene β‐cyclase (*LCYB*) gene. Mol. Breed. 20, 63–72.

[pbi13100-bib-0005] Barloy, D. , Lemoine, J. , Abelard, P. , Tanguy, A.M. , Rivoa, R. and Jahier, J. (2007) Marker‐assisted of two cereal cyst nematode resistance genes from *Aegilops variabilis* in wheat. Mol. Breed. 20, 31–40.

[pbi13100-bib-0006] Bedigian, D. (2003) Evolution of sesame revisited: domestication, diversity and prospects. Genet. Resour. Crop Evol. 50, 779–787.

[pbi13100-bib-0007] Bedigian, D. and Harlan, J.R. (1986) Evidence for cultivation of sesame in the ancient world. Econ. Bot. 40, 137–154.

[pbi13100-bib-0008] Benjamini, Y. and Hochberg, Y. (1995) Controlling the false discovery rate: a practical and powerful approach to multiple testing. J. R. Stat. Soc. Series B, 57, 289–300.

[pbi13100-bib-0009] Boureima, S. , Eyletters, M. , Diouf, M. , Diop, T.A. and Van Damme, P. (2011) Sensitivity of seed germination and seedling radicle growth to drought stress in sesame (*Sesamum indicum* L.). Res. J. Environ. Sci. 5, 557–564.

[pbi13100-bib-0010] Boureima, S. , Diouf, M. , Amoukou, A.I. and Van Damme, P. (2016) Screening for sources of tolerance to drought in sesame induced mutants: assessment of indirect selection criteria for seed yield. Int. J. Pure Appl. Biosci. 4, 45–60.

[pbi13100-bib-0011] Bradbury, P.J. , Zhang, Z. , Kroon, D.E. , Casstevens, T.M. , Ramdoss, Y. and Buckler, E.S. (2007) TASSEL: software for association mapping of complex traits in diverse samples. Bioinformatics, 23, 2633–2635. 17586829 10.1093/bioinformatics/btm308

[pbi13100-bib-0012] Cao, K. , Zhou, Z. , Wang, Q. , Guo, J. , Zhao, P. , Zhu, G. , Fang, W. *et al*. (2016) Genome‐wide association study of 12 agronomic traits in peach. Nat. Commun. 7, 13246. 27824331 10.1038/ncomms13246 PMC5105138

[pbi13100-bib-0013] Chowdhury, S. , Basu, A. and Kundu, S. (2017) Overexpression of a new Osmotin‐Like Protein gene (*SindOLP*) confers tolerance against biotic and abiotic stresses in sesame. Front. Plant Sci. 8, 410. 28400780 10.3389/fpls.2017.00410 PMC5368222

[pbi13100-bib-0014] Clough, S.J. and Bent, A.F. (1998) Floral dip, a simplified method for Agrobacterium‐ mediated transformation of *Arabidopsis thaliana* . Plant J. 16, 735–743. 10069079 10.1046/j.1365-313x.1998.00343.x

[pbi13100-bib-0015] Costaglioli, P. , Joubès, J. , Garcia, C. , Stef, M. , Arveiler, B. , Lessire, R. and Garbay, B. (2005) Profiling candidate genes involved in wax biosynthesis in *Arabidopsis thaliana* by microarray analysis. Biochim. Biophys. Acta, 1734, 247–258. 15914083 10.1016/j.bbalip.2005.04.002

[pbi13100-bib-0016] Dossa, K. (2016) A physical map of important QTLs, functional markers and genes available for sesame breeding programs. Physiol. Mol. Biol. Plants, 22, 613–619. 27924134 10.1007/s12298-016-0385-8 PMC5120042

[pbi13100-bib-0017] Dossa, K. , Wei, X. , Zhang, Y. , Fonceka, D. , Yang, W. , Diouf, D. , Liao, B. *et al*. (2016) Analysis of genetic diversity and population structure of sesame accessions from Africa and Asia as major centers of its cultivation. Genes (Basel), 7, 14. 27077887 10.3390/genes7040014 PMC4846844

[pbi13100-bib-0018] Dossa, K. , Diouf, D. , Wang, L. , Wei, X. , Zhang, Y. , Niang, M. , Fonceka, D. *et al*. (2017) The emerging oilseed crop *Sesamum indicum* enters the “Omics” era. Front. Plant Sci. 8, 1154. 28713412 10.3389/fpls.2017.01154 PMC5492763

[pbi13100-bib-0019] Dray, S. , Dufour, A. , Leeuw, J.D. and Zeileis, A. (2007) The ade4 package: implementing the duality diagram for ecologists. J. Stat. Softw. 22, 1–20.

[pbi13100-bib-0020] Duggal, P. , Gillanders, E.M. , Holmes, T.N. and Bailey‐Wilson, J.E. (2008) Establishing an adjusted p‐value threshold to control the family‐wide type 1 error in genome wide association studies. BMC Genom. 9, 516. 10.1186/1471-2164-9-516 PMC2621212 18976480

[pbi13100-bib-0021] Evanno, G. , Regnaut, S. and Goudet, J. (2005) Detecting the number of clusters of individuals using the software STRUCTURE: a simulation study. Mol. Ecol. 14, 2611–2620. 15969739 10.1111/j.1365-294X.2005.02553.x

[pbi13100-bib-0022] Falush, D. , Stephens, M. and Pritchard, J.K. (2003) Inference of population structure, extensions to linked loci and correlated allele frequencies. Genetics, 164, 1567–1587. 12930761 10.1093/genetics/164.4.1567 PMC1462648

[pbi13100-bib-0023] FAOSTAT , Food and Agriculture Organization statistical databases (2017). http://faostatfao.org/

[pbi13100-bib-0024] Farooq, M. , Wahid, A. , Kobayashi, N. , Fujita, D. and Basra, S.M.A. (2009) Plant drought stress: effects, mechanisms and management. Agron. Sustain. Dev. 29, 185–212.

[pbi13100-bib-0025] Finkelstein, R.R. , Wang, M.L. , Lynch, T.J. , Rao, S. and Goodman, H.M. (1998) The Arabidopsis abscisic acid response locus *ABI4* encodes an APETALA2 domain protein. Plant Cell, 10, 1043–1054. 9634591 10.1105/tpc.10.6.1043 PMC144030

[pbi13100-bib-0026] Gong, B. , Li, X. , VandenLangenberg, K.M. , Wen, D. , Sun, S. , Wei, M. , Li, Y. *et al*. (2014) Overexpression of S‐adenosyl‐L‐methionine synthetase increased tomato tolerance to alkali stress through polyamine metabolism. Plant Biotechnol. J. 12, 694–708. 24605920 10.1111/pbi.12173

[pbi13100-bib-0027] Götz, S. , García‐Gómez, J.M. , Terol, J. , Williams, T.D. , Nagaraj, S.H. , Nueda, M.J. , Robles, M. *et al*. (2008) High throughput functional annotation and data mining with the Blast2GO suite. Nucleic Acids Res. 36, 3420–3435. 18445632 10.1093/nar/gkn176 PMC2425479

[pbi13100-bib-0028] Guo, Z. , Yang, W. , Chang, Y. , Ma, X. , Tu, H. , Xiong, F. , Jiang, N. *et al*. (2018) Genome‐wide association studies of image traits reveal genetic architecture of drought resistance in rice. Mol. Plant, 11, 789–805. 29614319 10.1016/j.molp.2018.03.018

[pbi13100-bib-0029] Hall, T.A. (1999) BioEdit: a user‐friendly biological sequence alignment editor and analysis program for Windows 95/98/NT. Nucleic Acids Symp. Ser. 41, 95–98.

[pbi13100-bib-0030] Hardy, O.J. and Vekemans, X. (2002) SPAGeDi: a versatile computer program to analyse spatial genetic structure at the individual or population levels. Mol. Ecol. Notes, 2, 618–620.

[pbi13100-bib-0031] Hassanzadeh, M. , Asghari, A. , Jamaati‐e‐Somarin, S. , Saeidi, M. , Zabihi‐e‐Mahmoodabad, R. and Hokmalipour, S. (2009) Effects of water deficit on drought tolerance indices of sesame (*Sesamum indicum* L.) genotypes in moghan region. Res. J. Environ. Sci. 3, 116–121.

[pbi13100-bib-0032] Hiltebrandt, V.M. (1932) Sesame (*Sesamum indicum* L.). Bull. Appl. Bot. Plant Breed. 2, 1–114.

[pbi13100-bib-0033] Huang, X. and Han, B. (2014) Natural variations and genome‐wide association studies in crop plants. Annu. Rev. Plant Biol. 65, 531–551. 24274033 10.1146/annurev-arplant-050213-035715

[pbi13100-bib-0034] Huang, X. , Wei, X. , Sang, T. , Zhao, Q. , Feng, Q. , Zhao, Y. , Li, C. *et al*. (2010) Genome‐wide association studies of 14 agronomic traits in rice landraces. Nat. Genet. 42, 961–967. 20972439 10.1038/ng.695

[pbi13100-bib-0035] Huang, X. , Zhao, Y. , Wei, X. , Li, C. , Wang, A. , Zhao, Q. , Li, W. *et al*. (2012) Genome‐wide association study of flowering time and grain yield traits in a worldwide collection of rice germplasm. Nat. Genet. 44, 32–39. 10.1038/ng.1018 22138690

[pbi13100-bib-0036] Islam, F. , Gill, R.A. , Ali, B. , Farooq, M.A. , Xu, L. , Najeeb, U. and Zhou, W. (2016). Sesame. In Breeding Oilseed Crop for Sustainable Production: Opportunities and Constraints ( Gupta, S.K. , ed.), pp. 135–147. Cambridge, MA: Academic Press.

[pbi13100-bib-0037] Juenger, T.E. (2013) Natural variation and genetic constraints on drought tolerance. Curr. Opin. Plant Biol. 16, 274–281. 23462639 10.1016/j.pbi.2013.02.001

[pbi13100-bib-0038] Kang, H.M. , Sul, J.H. , Service, S.K. , Zaitlen, N.A. , Kong, S. , Freimer, N.B. , Sabatti, C. *et al*. (2010) Variance component model to account for sample structure in genome‐wide association studies. Nat. Genet. 42, 348–354. 20208533 10.1038/ng.548 PMC3092069

[pbi13100-bib-0039] Kang, Y. , Sakiroglu, M. , Krom, N. , Stanton‐Geddes, J. , Wang, M. , Lee, Y.‐C. , Young, N.D. *et al*. (2015) Genome‐wide association of drought‐related and biomass traits with HapMap SNPs in *Medicago truncatula* . Plant, Cell Environ. 38, 1997–2011. 25707512 10.1111/pce.12520

[pbi13100-bib-0040] Kassab, O.M. , Mehanna, H.M. and Aboelill, A. (2012) Drought impact on growth and yield of some sesame varieties. J. Appl. Sci. Res. 8, 4544–4551.

[pbi13100-bib-0041] Kende, H. (1993) Ethylene biosynthesis. Annu. Rev. Plant Physiol. Plant Mol. Biol. 44, 283–307.

[pbi13100-bib-0042] Kim, S.H. , Kim, S.H. , Palaniyandi, S.A. , Yang, S.H. and Suh, J.‐W. (2015) Expression of potato S‐adenosyl‐L‐methionine synthase (*SbSAMS*) gene altered developmental characteristics and stress responses in transgenic *Arabidopsis* plants. Plant Physiol. Biochem. 87, 84–91. 25559387 10.1016/j.plaphy.2014.12.020

[pbi13100-bib-0043] Kowles, R. (2001) Solving Problems in Genetics. New York, NY: Springer‐Verlag.

[pbi13100-bib-0044] Kumar, S. , Stecher, G. and Tamura, K. (2016) MEGA7: Molecular Evolutionary Genetics Analysis Version 7.0 for bigger datasets. Mol. Biol. Evol. 33, 1870–1874. 27004904 10.1093/molbev/msw054 PMC8210823

[pbi13100-bib-0045] Lalitha, S. (2000) Primer premier 5. Biotech Softw. Internet Rep. 1, 270–272.

[pbi13100-bib-0046] Lam, H.M. , Xu, X. , Liu, X. , Chen, W. , Yang, G. , Wong, F.L. , Li, M.W. *et al*. (2010) Resequencing of 31 wild and cultivated soybean genomes identifies patterns of genetic diversity and selection. Nat. Genet. 42, 1053–1059. 21076406 10.1038/ng.715

[pbi13100-bib-0047] Langham, R. (2007) Phenology of sesame. In Issues in New Crop and New Uses ( Janick, J. and Whipkey, A. , eds),. Virginia: ASHS Press, Alexandria.

[pbi13100-bib-0048] Lescot, M. , Déhais, P. , Moreau, Y. , De Moor, B. , Rouzé, P. and Rombauts, S. (2002) PlantCARE: a database of plant cis‐acting regulatory elements and a portal to tools for in silico analysis of promoter sequences. Nucleic Acids Res. 30, 325–327. 11752327 10.1093/nar/30.1.325 PMC99092

[pbi13100-bib-0049] Li, M.X. , Yeung, J.M.Y. , Cherny, S.S. and Sham, P.C. (2012) Evaluating the effective numbers of independent tests and significant p‐value thresholds in commercial genotyping arrays and public imputation reference datasets. Hum. Genet. 131, 747–756. 22143225 10.1007/s00439-011-1118-2 PMC3325408

[pbi13100-bib-0050] Li, H. , Peng, Z. , Yang, X. , Wang, W. , Fu, J. , Wang, J. , Han, Y. *et al*. (2013) Genome‐wide association study dissects the genetic architecture of oil biosynthesis in maize kernels. Nat. Genet. 45, 43–50. 23242369 10.1038/ng.2484

[pbi13100-bib-0051] Li, L. , Luo, Y. , Chen, B. , Xu, K. , Zhang, F. , Li, H. , Huang, Q. *et al*. (2016) A Genome‐wide association study reveals new loci for resistance to clubroot disease in *Brassica napus* . Front. Plant Sci. 7, 1483. 27746804 10.3389/fpls.2016.01483 PMC5044777

[pbi13100-bib-0052] Lin, F. , Wang, S. , Hu, Y. and He, B. (2008) Cloning of a S‐Adenosylmethionine synthetase gene from broomcorn millet (*Panicum miliaceum* L.) and its expression during drought and re‐watering. Acta Agron. Sin. 34, 777–782.

[pbi13100-bib-0053] Liu, S. , Wang, X. , Wang, H. , Xin, H. , Yang, X. , Yan, J. , Li, J. *et al*. (2013) Genome‐wide analysis of *ZmDREB* genes and their association with natural variation in drought tolerance at seedling stage of *Zea mays* L. PLoS Genet. 9, e1003790. 24086146 10.1371/journal.pgen.1003790 PMC3784558

[pbi13100-bib-0054] Livak, K.J. and Schmittgen, T.D. (2001) Analysis of relative gene expression data using real‐time quantitative PCR and the 2^−∆∆Ct^ method. Methods, 25, 402–408. 11846609 10.1006/meth.2001.1262

[pbi13100-bib-0055] Long, N.V. , Dolstra, O. , Malosetti, M. , Kilian, B. , Graner, A. , Visser, R.G.F. and van der Linden, C.G. (2013) Association mapping of salt tolerance in barley (*Hordeum vulgare* L.). Theor. Appl. Genet. 126, 2335–2351. 23771136 10.1007/s00122-013-2139-0

[pbi13100-bib-0056] Lu, K. , Peng, L. , Zhang, C. , Lu, J. , Yang, B. , Xiao, Z. , Liang, Y. *et al*. (2017) Genome‐wide association and transcriptome analyses reveal candidate genes underlying yield‐determining traits in *Brassica napus* . Front. Plant Sci. 8, 206. 28261256 10.3389/fpls.2017.00206 PMC5309214

[pbi13100-bib-0057] Luna, A. and Nicodemus, K.K. (2007) snp.plotter: an R‐based SNP/haplotype association and linkage disequilibrium plotting package. Bioinformatics, 23, 774–776. 17234637 10.1093/bioinformatics/btl657

[pbi13100-bib-0058] de Mendiburu, F. (2014) Agricolae: statistical procedures for agricultural research. *R package * **1**: 1–16.

[pbi13100-bib-0059] Moeder, W. , Garcia‐Petit, C. , Ung, H. , Fucile, G. , Samuel, M.A. , Christendat, D. and Yoshioka, K. (2013) Crystal structure and biochemical analyses reveal that the Arabidopsis triphosphate tunnel metalloenzyme *AtTTM3* is a tripolyphosphatase involved in root development. Plant J. 76, 615–626. 24004165 10.1111/tpj.12325

[pbi13100-bib-0060] Morris, G.P. , Ramu, P. , Deshpande, S.P. , Hash, C.T. , Shah, T. , Upadhyaya, H.D. , Riera‐Lizarazu, O. *et al*. (2012) Population genomic and genome‐wide association studies of agroclimatic traits in sorghum. Proc. Natl Acad. Sci. USA, 110, 453–458. 23267105 10.1073/pnas.1215985110 PMC3545811

[pbi13100-bib-0061] Nordborg, M. and Weigel, D. (2008) Next‐generation genetics in plants. Nature, 456, 720–723. 19079047 10.1038/nature07629

[pbi13100-bib-0062] Pál, M. , Szalai, G. and Janda, T. (2015) Speculation: polyamines are important in abiotic stress signaling. Plant Sci. 237, 16–23. 26089148 10.1016/j.plantsci.2015.05.003

[pbi13100-bib-0063] R Development Core Team (2015) R: A Language and Environment for Statistical Computing. Vienna, Austria: R Foundation for Statistical Computing.

[pbi13100-bib-0064] Radadiya, N. , Parekh, V.B. , Dobariya, B. , Mahatma, L. and Mahatma, M.K. (2016) Abiotic stresses alter expression of *S‐Adenosylmethionine synthetase* gene, polyamines and antioxidant activity in pigeon pea (*Cajanus cajan* L.). Legume Res. 39, 905–913.

[pbi13100-bib-0065] Rozas, J. , Ferrer‐Mata, A. , Sánchez‐Delbarrio, J.C. , Guirao‐Rico, S. , Librado, P. , Ramos‐Onsins, S.E. and Sánchez‐Gracia, A. (2017) DnaSP v6: DNA sequence polymorphism analysis of large datasets. Mol. Biol. Evol. 34, 3299–3302. 29029172 10.1093/molbev/msx248

[pbi13100-bib-0066] Sacco, A. , Di, M.A. , Lombardi, N. , Trotta, N. , Punzo, B. , Mari, A. and Barone, A. (2013) Quantitative trait loci pyramiding for fruit quality traits in tomato. Mol. Breed. 31, 217–222. 23316114 10.1007/s11032-012-9763-2 PMC3538004

[pbi13100-bib-0067] Su, J. , Pang, C. , Wei, H. , Li, L. , Liang, B. , Wang, C. , Song, M. *et al*. (2016) Identification of favorable SNP alleles and candidate genes for traits related to early maturity via GWAS in upland cotton. BMC Genom. 17, 687. 10.1186/s12864-016-2875-z PMC5006539 27576450

[pbi13100-bib-0068] Sun, J. , Rao, Y. , Le, M. , Yan, T. , Yan, X. and Zhou, H. (2010) Effects of drought stress on sesame growth and yield characteristics and comprehensive evaluation of drought tolerance. Chin. J. Oil Crop Sci. 32, 525–533.

[pbi13100-bib-0069] Tardieu, F. , Cabrera‐Bosquet, L. , Pridmore, T. and Bennett, M. (2017) Plant phenomics, from sensors to knowledge. Curr. Biol. 27, R770–R783. 28787611 10.1016/j.cub.2017.05.055

[pbi13100-bib-0070] Tian, F. , Bradbury, P.J. , Brown, P.J. , Hung, H. , Sun, Q. , Flint‐Garcia, S. , Rocheford, T.R. *et al*. (2011) Genome‐wide association study of leaf architecture in the maize nested association mapping population. Nat. Genet. 43, 159–162. 21217756 10.1038/ng.746

[pbi13100-bib-0071] Tiburcio, A.F. , Kaur‐Sawhney, R. and Galston, A.W. (1990). Polyamine metabolism. In:Intermedatory Nitrogen Metabolism.16, the Biochemistry of Plants ( Miflin, B.J. and Lea, P.J. , eds), pp. 283–325. Cambridge, MA: Academic Press.

[pbi13100-bib-0072] Turner, S.D. (2014) Qqman: an R package for visualizing GWAS results using Q‐Q and Manhattan pots. BioRxiv, 10.1101/005165.

[pbi13100-bib-0073] Wan, H. , Chen, L. , Guo, J. , Li, Q. , Wen, J. , Yi, B. , Ma, C. *et al*. (2017) Genome‐wide association study reveals the genetic architecture underlying salt tolerance‐related traits in rapeseed (*Brassica napus* L.). Front. Plant Sci. 8, 593. 28491067 10.3389/fpls.2017.00593 PMC5405135

[pbi13100-bib-0074] Wang, L. , Zhang, Y. , Li, P. , Wang, X. , Zhang, W. , Wei, W. and Zhang, X. (2012) HPLC analysis of seed sesamin and sesamolin variation in a sesame germplasm collection in China. J. Am. Oil Chem. Soc. 8, 1011–1020.

[pbi13100-bib-0075] Wang, L. , Yu, S. , Tong, C. , Zhao, Y. , Liu, Y. , Song, C. , Zhang, Y. *et al*. (2014) Genome sequencing of the high oil crop sesame. Genome Biol. 15, R39. 24576357 10.1186/gb-2014-15-2-r39 PMC4053841

[pbi13100-bib-0076] Wang, X. , Oh, M.W. and Komatsu, S. (2016a) Characterization of S‐adenosylmethionine synthetases in soybean under flooding and drought stresses. Biol. Plant. 60, 269.

[pbi13100-bib-0077] Wang, X. , Wang, H. , Liu, S. , Ferjani, A. , Li, J. , Yan, J. , Yang, X. *et al*. (2016b) Genetic variation in *ZmVPP1* contributes to drought tolerance in maize seedlings. Nat. Genet. 48, 1233–1241. 27526320 10.1038/ng.3636

[pbi13100-bib-0078] Wei, X. , Liu, K. , Zhang, Y. , Feng, Q. , Wang, L. , Zhao, Y. , Li, D. *et al*. (2015) Genetic discovery for oil production and quality in sesame. Nat. Commun. 6, 8609. 26477832 10.1038/ncomms9609 PMC4634326

[pbi13100-bib-0079] Wei, X. , Gong, H. , Yu, J. , Liu, P. , Wang, L. , Zhang, Y. and Zhang, X. (2017) Sesame FG: an integrated database for the functional genomics of sesame. Sci. Rep. 7, 2342. 28539606 10.1038/s41598-017-02586-3 PMC5443765

[pbi13100-bib-0080] Werner, K. , Friedt, W. and Ordon, F. (2005) Strategies for pyramiding resistance genes against the barley yellow mosaic virus complex (BaMMV, BaYMV, BaYMV‐2). Mol. Breed. 16, 45–55.

[pbi13100-bib-0081] Witcombe, J.R. , Hollington, P.A. , Howarth, C.J. , Reader, S. and Steele, K.A. (2007) Breeding for abiotic stresses for sustainable agriculture. Philos. Trans. R. Soc. B 363, 703–716. 10.1098/rstb.2007.2179 PMC2610105 17761467

[pbi13100-bib-0082] Ye, J. , Fang, L. , Zheng, H. , Zhang, Y. , Chen, J. , Zhang, Z. , Wang, J. *et al*. (2006) WEGO: a web tool for plotting GO annotations. Nucleic Acids Res. 34, W293–W297. 16845012 10.1093/nar/gkl031 PMC1538768

[pbi13100-bib-0083] Zhang, Z. , Ersoz, E. , Lai, C.Q. , Todhunter, R.J. , Tiwari, H.K. , Gore, M.A. , Bradbury, P.J. *et al*. (2010) Mixed linear model approach adapted for genome‐wide association studies. Nat. Genet. 42, 355–360. 20208535 10.1038/ng.546 PMC2931336

[pbi13100-bib-0084] Zhang, B. , Li, W. , Chang, X. , Li, R. and Jing, R. (2014) Effects of favorable alleles for water soluble carbohydrates at grain filling on grain weight under drought and heat stresses in wheat. PLoS ONE, 29, e102917. 10.1371/journal.pone.0102917 PMC4103880 25036550

[pbi13100-bib-0085] Zwicker, S. , Mast, S. , Stos, V. , Pfitzner, A.J.P. and Pfitzner, U.M. (2007) Tobacco NIMIN2 proteins control PR gene induction through transient repression early in systemic acquired resistance. Mol. Plant Pathol. 8, 385–400. 20507508 10.1111/j.1364-3703.2007.00399.x

