# Supplementary figures and images for: The genetic basis of drought tolerance in the high oil crop Sesamum indicum
Source: Plant Biotechnol J. 2019 Mar 5;17(9):1788–803. doi: 10.1111/pbi.13100 (PMC6686131; doi:10.1111/pbi.13100)

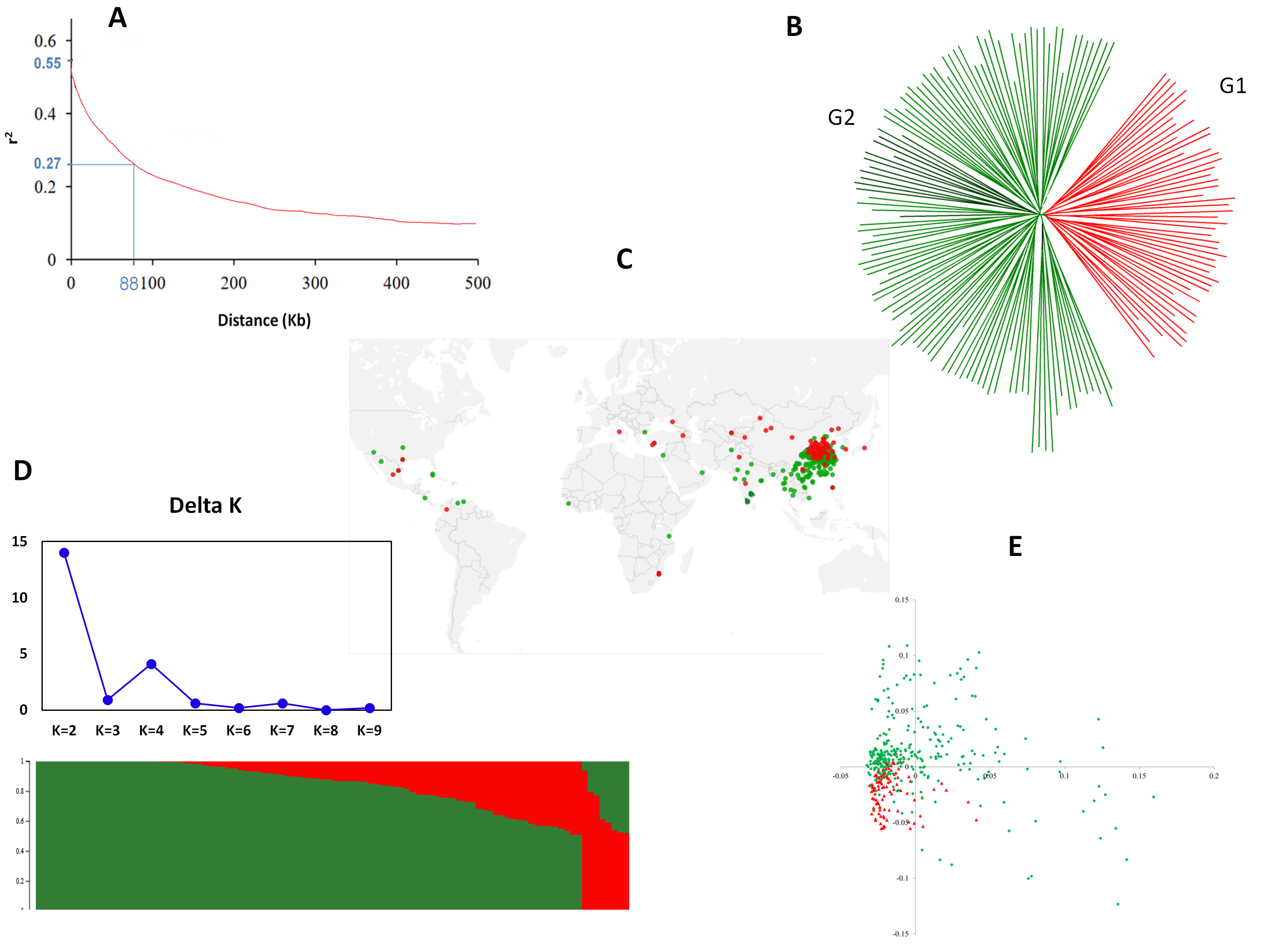

Supplement: Supplementary file 1 — Figure S1 Population genetics of the Sesamum indicum association panel. [file PBI-17-1788-s001.tif]

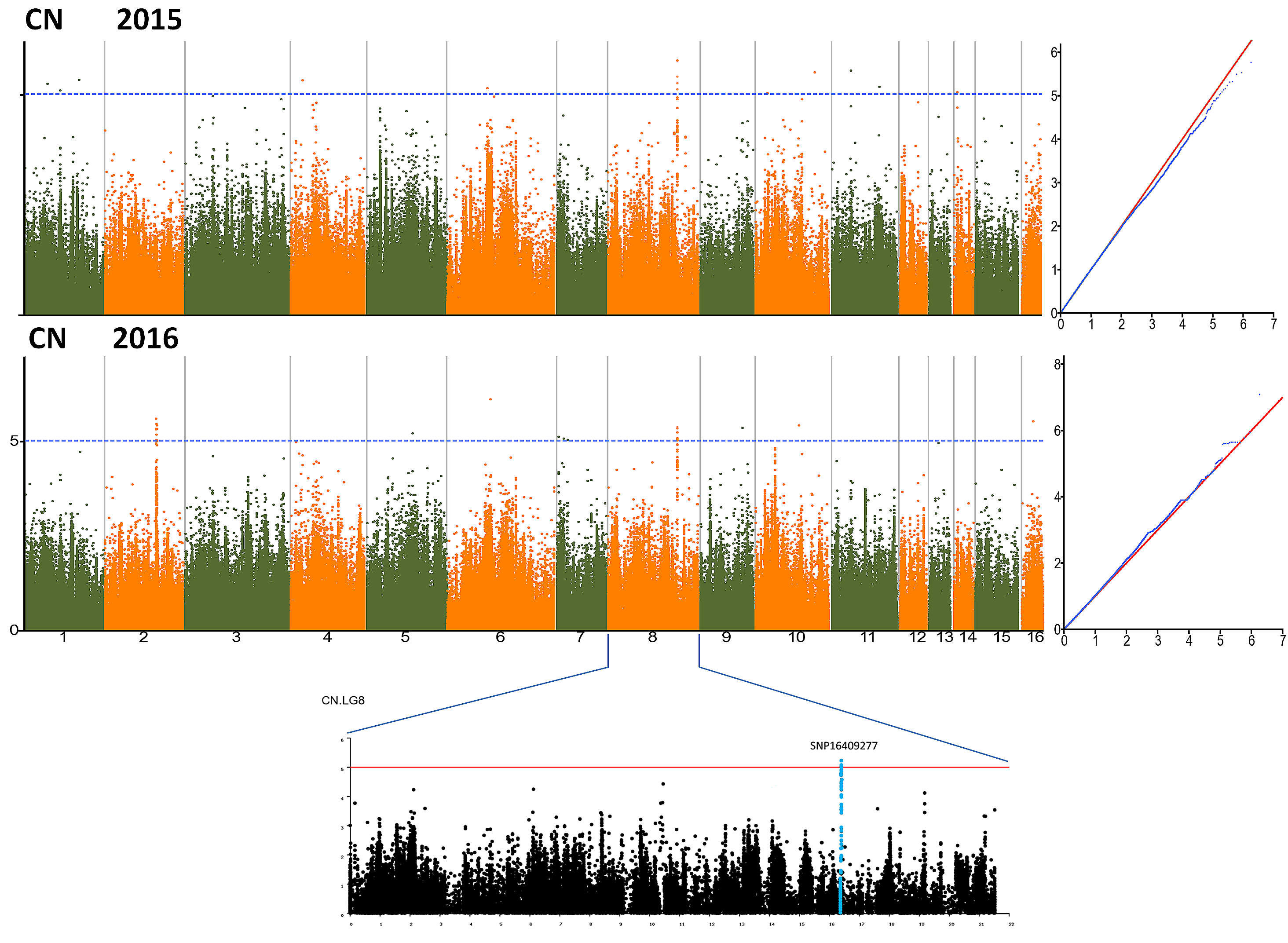

Supplement: Supplementary file 2 — Figure S2 Manhattan plots and QQ plots of genome‐wide association studies using the mixed model for relative capsule number (CN) in Sesamum indicum during 2015 and 2016. [file PBI-17-1788-s016.tif]

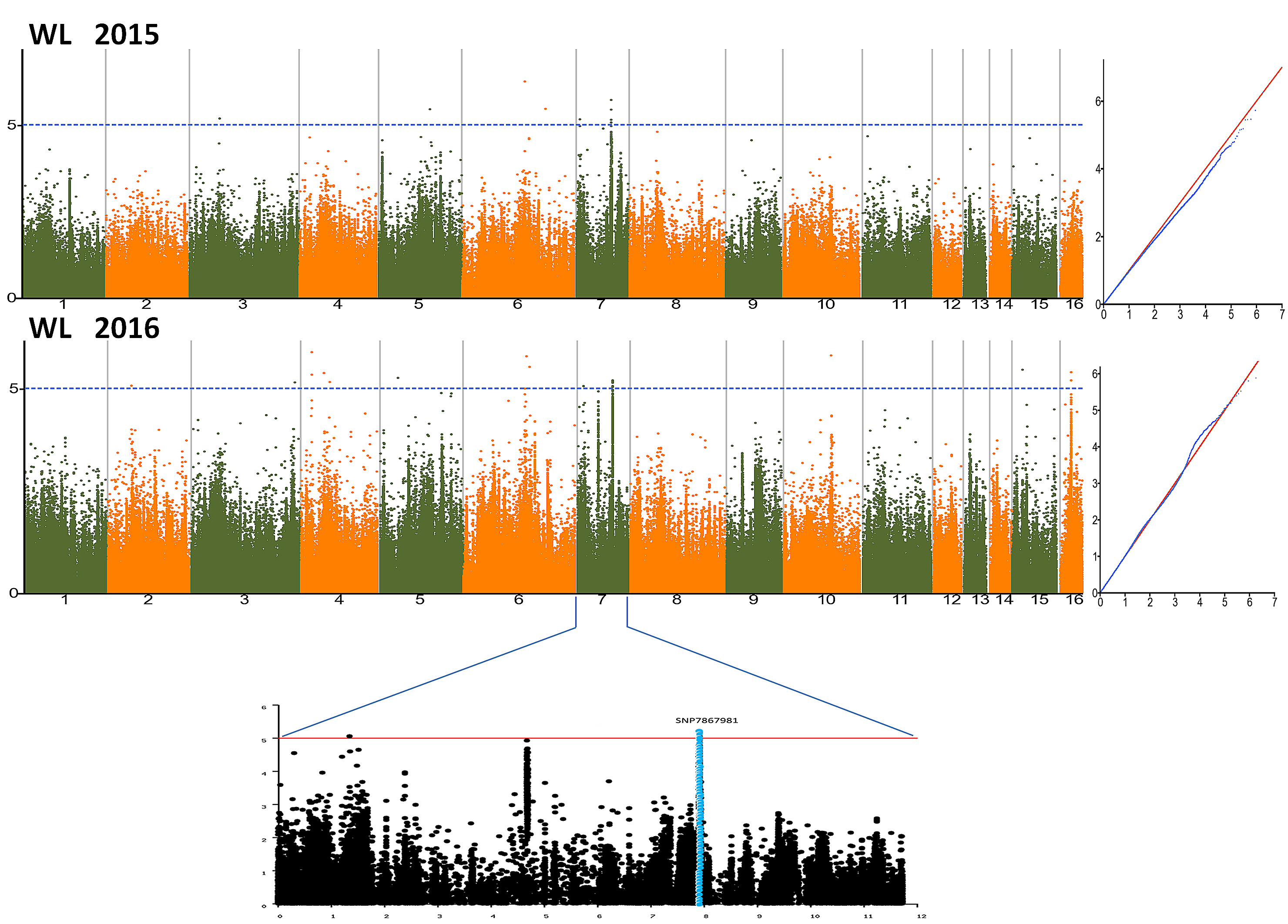

Supplement: Supplementary file 3 — Figure S3 Manhattan plots and QQ plots of genome‐wide association studies using the Mixed model for Wiling level (WL) in Sesamum indicum during 2015 and 2016. [file PBI-17-1788-s015.tif]

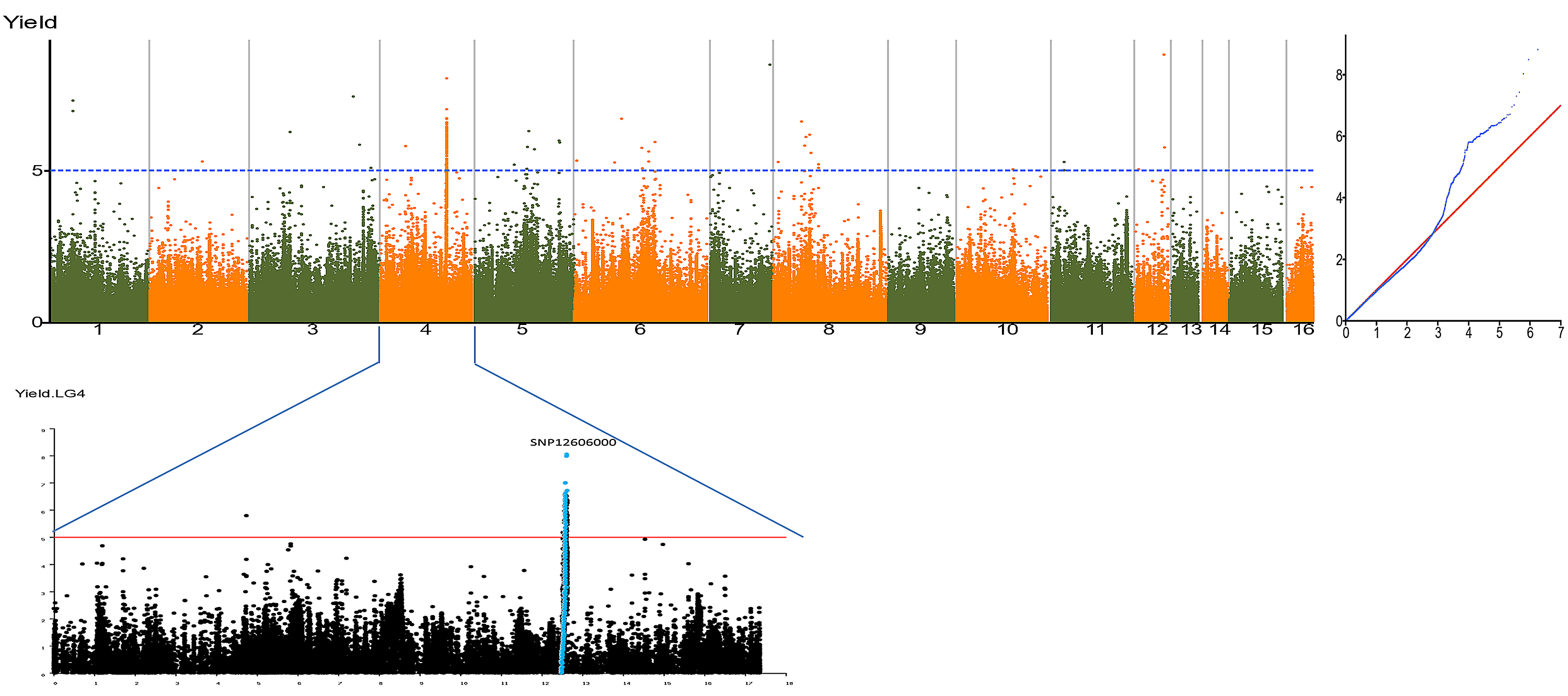

Supplement: Supplementary file 4 — Figure S4 Manhattan plots and QQ plots of genome‐wide association studies using the Mixed model for relative seed yield (Yie) trait in Sesamum indicum during 2016. [file PBI-17-1788-s017.tif]

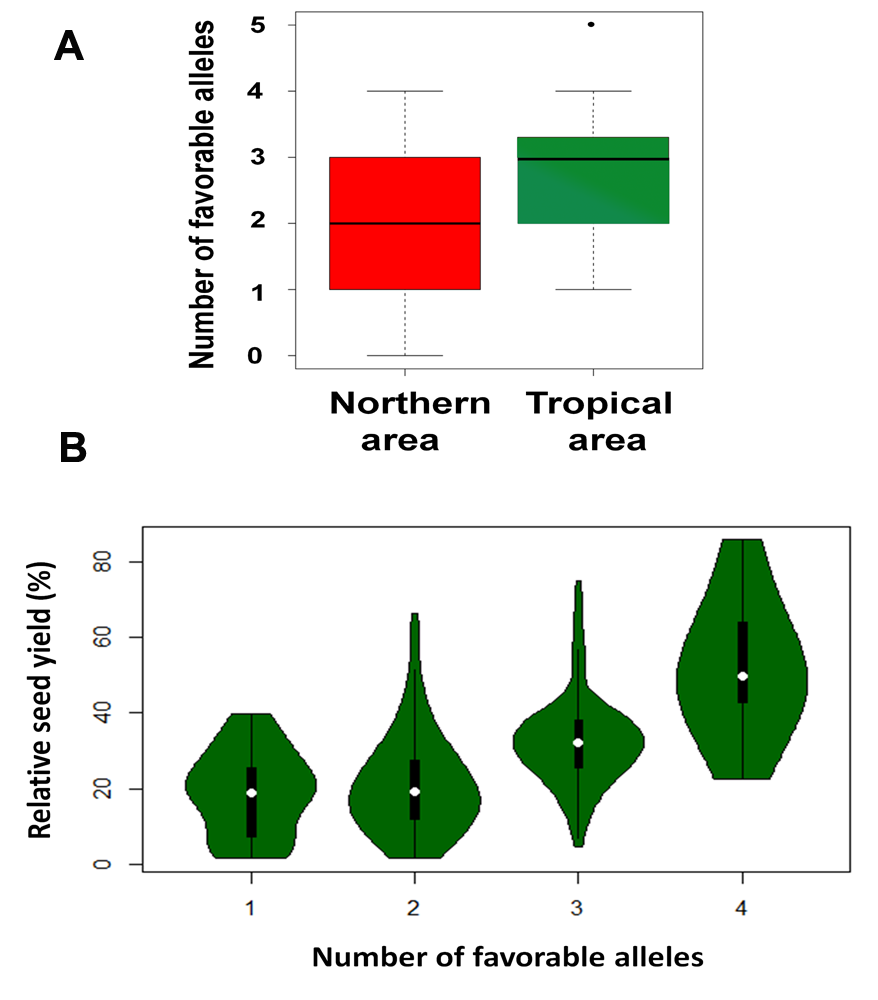

Supplement: Supplementary file 5 — Figure S5 (A) Boxplot displaying the number of favourable alleles between the two groups (tropical and northern areas) of Sesamum indicum accessions; (B) Pyramiding of favourable alleles at the loci SNP12606000, SNP9732360, SNP16406525 and SNP7867981 improves the seed yield maintenance of sesame accessions under drought stress. [file PBI-17-1788-s018.tif]

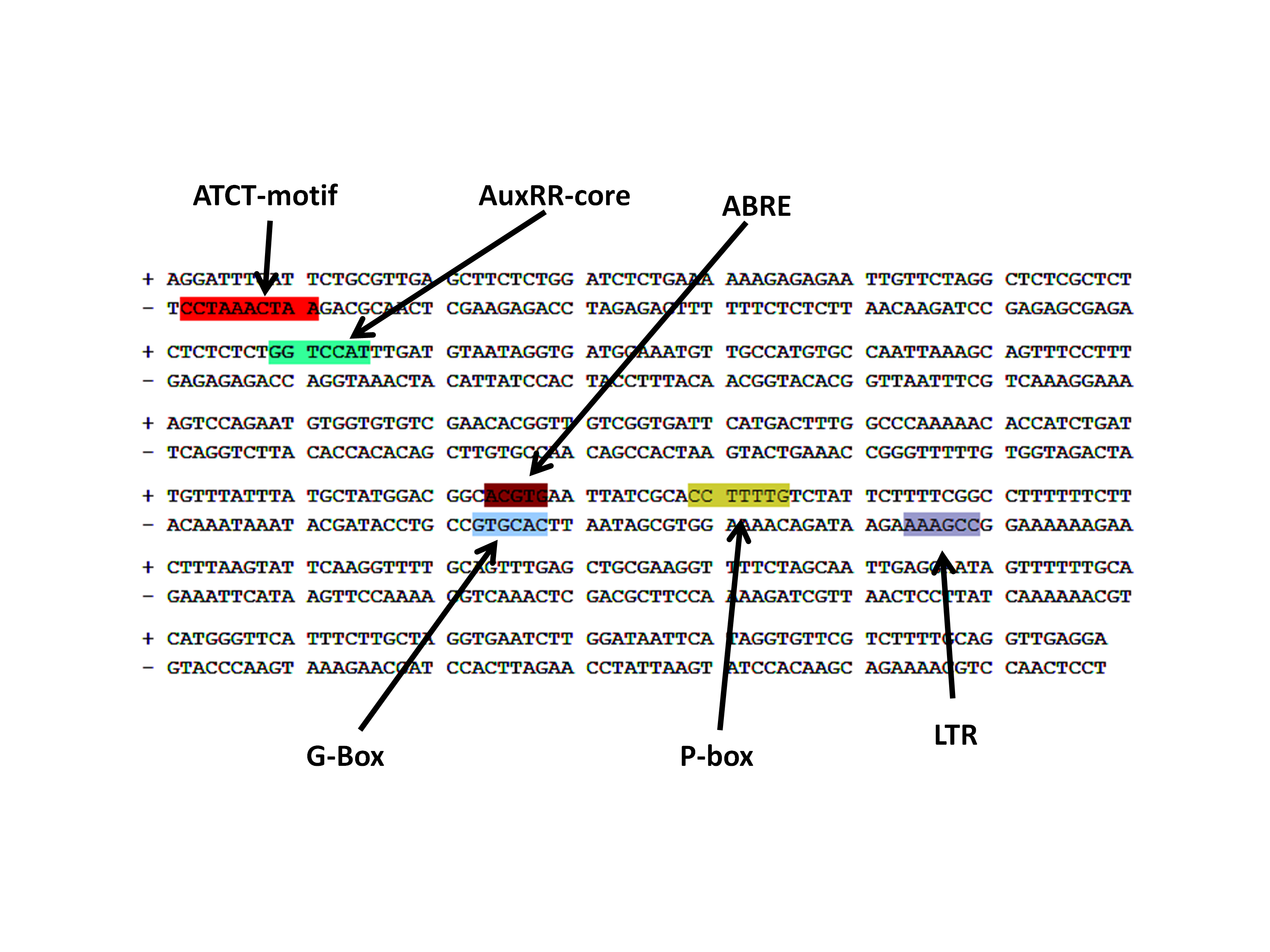

Supplement: Supplementary file 6 — Figure S6 Cis‐acting regulatory elements detected in the promoter of SiSAM. [file PBI-17-1788-s002.tif]

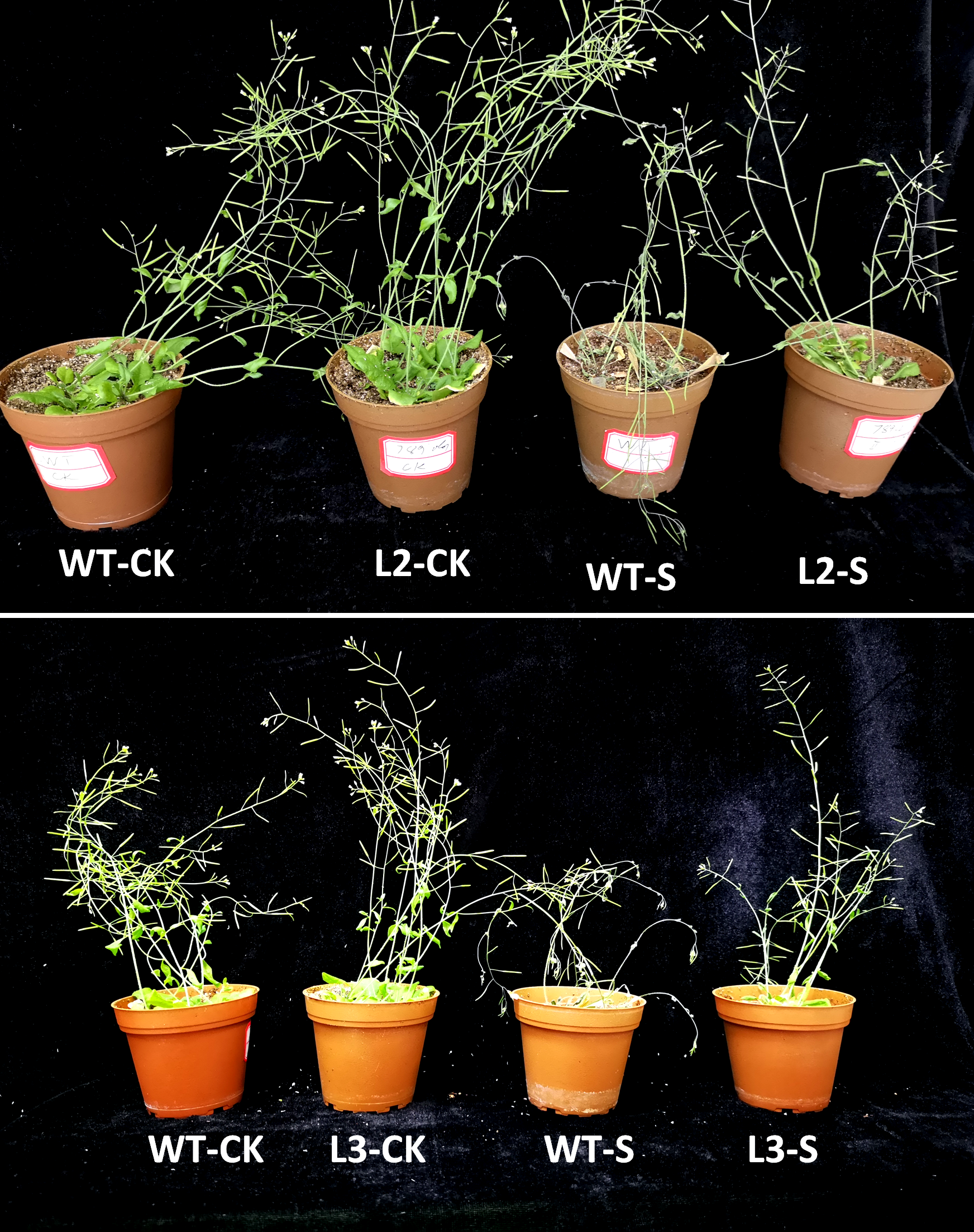

Supplement: Supplementary file 7 — Figure S7 Phenotypes of wild type (WT) and transgenic Arabidopsis thaliana plants (L2‐L3) over‐expressing SiSAM C , after 17 days water stress (S) and normal conditions (CK). [file PBI-17-1788-s003.tif]

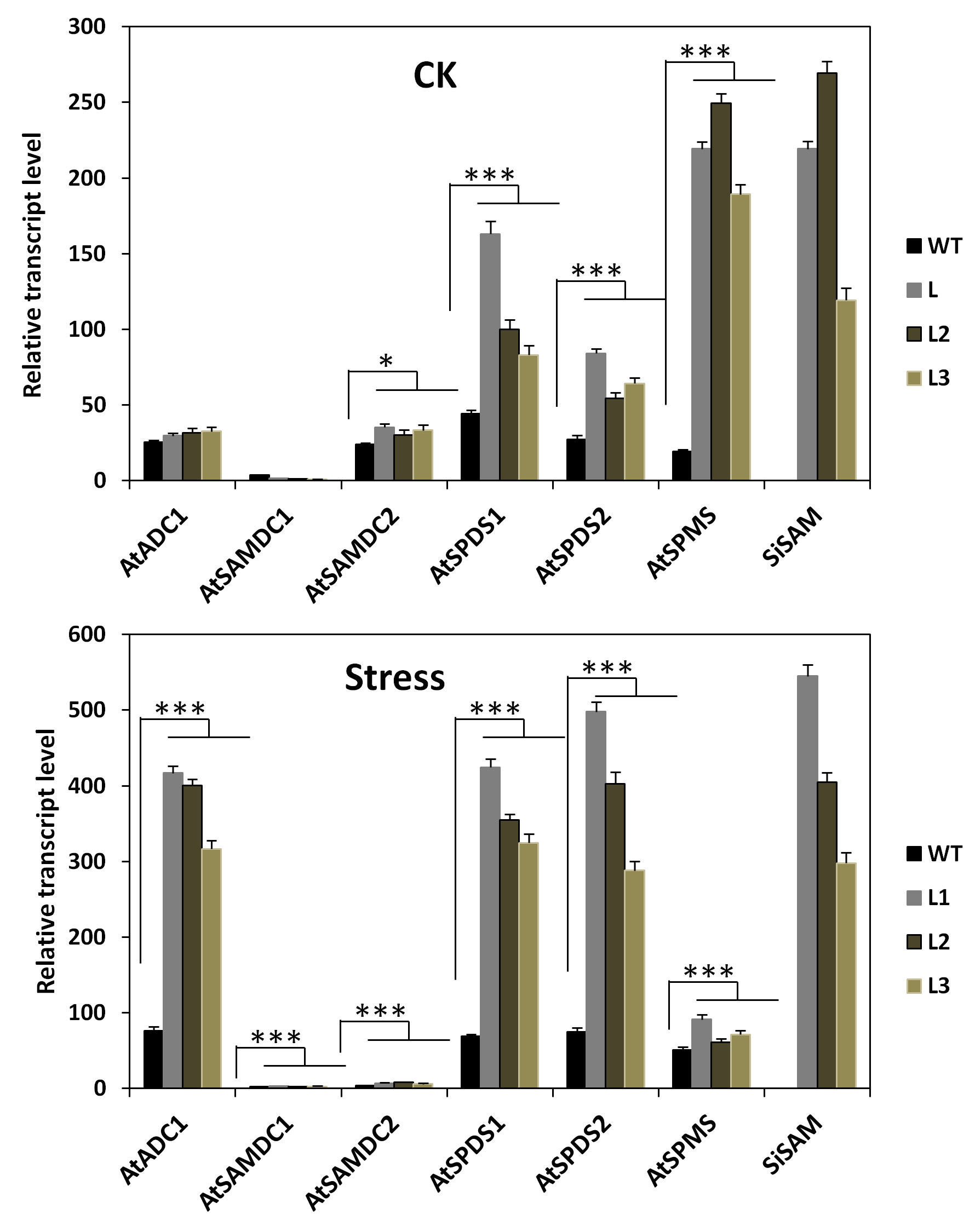

Supplement: Supplementary file 8 — Figure S8 Effects of drought stress on relative transcript levels of polyamine metabolism and biosynthesis key genes: SiSAM C , AtADC, AtSAMDC, AtSPDS and AtSPMS in wild type (WT) plants and transgenic Arabidopsis thaliana lines (L1, L2 and L3) over‐expressing SiSAM C . [file PBI-17-1788-s004.tif]

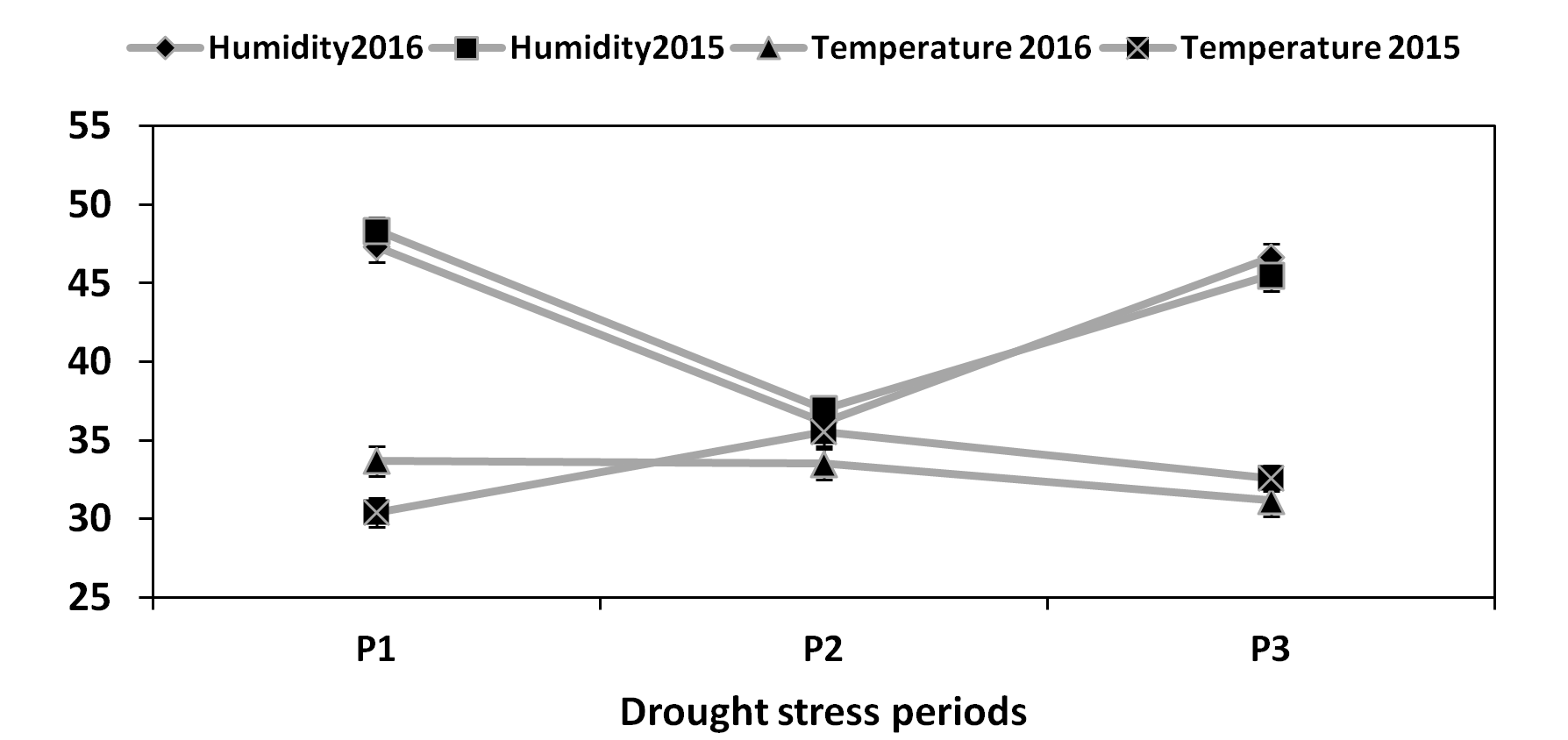

Supplement: Supplementary file 9 — Figure S9 Variation of the mean temperature and relative humidity during repeated drought treatment periods on Sesamum indicum accessions. [file PBI-17-1788-s005.tif]
